# Immune suppression of vaccine-induced CD8^+^ T-cell responses by gamma retrovirus envelope is mediated by interleukin-10-producing CD4^+^ T cells

**DOI:** 10.3389/fimmu.2022.934399

**Published:** 2022-12-20

**Authors:** Philip Podschwadt, Anna Malyshkina, Sonja Windmann, Athanasios Papadamakis, Leonie Kerkmann, Dennis Lapuente, Matthias Tenbusch, Mengji Lu, Michael Schindler, Karl Sebastian Lang, Wiebke Hansen, Wibke Bayer

**Affiliations:** ^1^ Institute for Virology, University Hospital Essen, University Duisburg-Essen, Essen, Germany; ^2^ Institute of Clinical and Molecular Virology, University Hospital Erlangen, Friedrich-Alexander University Erlangen-Nürnberg, Erlangen, Germany; ^3^ Department for Molecular Virology, Institute for Medical Virology and Epidemiology of Viral Diseases, University Hospital Tübingen, University Tübingen, Tübingen, Germany; ^4^ Institute for Immunology, University Hospital Essen, University Duisburg-Essen, Essen, Germany; ^5^ Institute for Medical Microbiology, University Hospital Essen, University Duisburg-Essen, Essen, Germany

**Keywords:** retrovirus, envelope, Env, Friend virus, immunosuppression, interleukin-10, IL-10

## Abstract

Retroviral envelope (Env) proteins have long been recognized to exhibit immunosuppressive properties, which affect the CD8^+^ T-cell response to an infection but also to immunization. Interestingly, we previously showed in the Friend murine leukemia virus (F-MuLV) model that the surface Env protein gp70 also plays a role in immunosuppression, in addition to the immunosuppressive function attributed to the transmembrane Env protein. We now demonstrate that immunization with F-MuLV Env leads to a significant increase in interleukin-10 (IL-10)-producing CD4^+^ T cells and that the induction of CD8^+^ T-cell responses in the presence of Env is rescued if the capacity of CD4^+^ T cells to produce IL-10 is abrogated, indicating a mechanistic role of IL-10-producing CD4^+^ T cells in mediating the Env-induced suppression of CD8^+^ T-cell responses in Env co-immunization. We found that CD8^+^ T-cell responses against different immunogens are not all equally affected. On the other hand, suppression of immunity was observed not only in co-immunization experiments but also for immune control of subcutaneous tumor growth after an Env immunization. Finally, we show that suppression of CD8^+^ T cells by the surface Env protein is observed not only for Friend MuLV Env but also for the Env proteins of other gamma retroviruses. Taken together, our results show that IL-10-producing CD4^+^ T cells mechanistically underlie the Env-mediated suppression of CD8^+^ T-cell responses and suggest the presence of an immunosuppressive motif in the surface Env protein of gamma retroviruses.

## Introduction

Retrovirus infections are associated with immunosuppression, which is mediated by different mechanisms. On the one hand, immunosuppression mediated by retroviruses is the inhibition of lymphoproliferative responses associated with altered cytokine profiles with increased interleukin (IL)-6 and IL-10 levels and decreased IL-2 levels (1). These changes in cytokine expression patterns are often attributed to the effects of retrovirus envelope (Env) proteins, which have long been recognized to have immunosuppressive properties. Early studies showed that inactivated particles or envelope proteins of a wide range of different retroviruses led to a suppression of mitogen-induced lymphocyte proliferation, cytokine production, and cytotoxicity [reviewed in (2)].

The retrovirus envelope protein is expressed as a polyprotein precursor that is proteolytically cleaved into the membrane-anchored transmembrane protein (TM), which contains the fusion peptide, and a surface (SU) protein that contains the receptor-binding domain (3). Early on, the TM Env has drawn special attention with regard to its immunosuppressive properties, whereas Env SU did not seem to contribute to the effects observed in suppression assays. A short peptide sequence in the Env TM protein has since been identified that seems to be responsible for the immunosuppression observed for Env TM proteins. This so-called immunosuppressive domain (ISD) has been shown to inhibit lymphocyte function (4), and *in-vitro* stimulation of PBMCs with the corresponding peptide was shown to induce a change in cytokine expression patterns, with inhibition of IL-2 and induction of IL-10 (5). It is hypothesized that the presence of this ISD in mammalian syncytin proteins, which are appropriated Env proteins of endogenized retroviruses, is crucial for the induction of an immunosuppressive, tolerogenic milieu in placental tissue (6, 7), even though direct evidence for immunosuppression by syncytins in the physiological setting is still missing. The immunosuppression mediated by Env is very strong and has been shown to allow engraftment of subcutaneous tumors in allogeneic mice when tumor cells were engineered to express whole Moloney murine leukemia virus Env, Env TM, or ISD alone (8). Interestingly, similar experiments with the Env of Mason-Pfizer monkey virus showed residual immunosuppression by Env even after deletion of the ISD, hinting at the presence of further immunosuppressive sequences (9).

Another mechanism that contributes to immune suppression in retrovirus infections is the expansion of regulatory T cells (Tregs), as has been analyzed in detail in the Friend virus (FV) model. It was shown that in FV infection, self-antigen-specific thymus-derived Tregs of broad specificities, as suggested by a broad distribution of T-cell receptor variable β chain usage (10), expand by one of two mechanisms. In an IL-2-dependent manner of activation, Tregs receive an activating signal from sensing increased levels of IL-2 produced by FV-specific CD4^+^ T cells *via* the IL-2 receptor molecule CD25, which in conjunction with self-antigen stimulation and glucocorticoid-induced TNF receptor-related protein (GITR)–GITR ligand interaction leads to Treg activation (10–12). In an IL-2-independent mechanism, Tregs that are specific for a superantigen of the endogenous retrovirus mouse mammary tumor virus 9 receive a secondary activating signal of membrane-bound tumor necrosis factor α (TNFα) on FV-specific CD8^+^ T cells *via* TNF receptor 2 (10, 12). An increase in Treg frequencies has also been described in humans infected with HIV; although the mechanism is not yet clear, the induction of semi-mature dendritic cells (DCs) by HIV has been shown to enhance Treg differentiation from conventional CD4^+^ T cells (13, 14).

Immunosuppression by retroviral Env proteins is relevant not only in retrovirus infection but of course also when retroviral Env proteins are used for immunization. In DNA immunization experiments, we showed that the co-immunization with plasmids encoding Friend murine leukemia virus (F-MuLV) Env and Leader-Gag resulted in a loss of CD8^+^ T-cell responses to the Leader-Gag-derived epitope GagL_85-93_. Interestingly, the induction of CD8^+^ T-cell responses could be rescued by co-immunization with plasmids encoding IL-2, IL-12, IL-21, IL-28A, and especially GM-CSF (15), showing that changes in the cytokine milieu influence the effect of Env on CD8^+^ T-cell responses. Interestingly, in adenovirus-based immunization experiments in the FV model, we demonstrated that immunosuppression was not only observed when the Env TM protein was included in the Env vaccine but also when only Env SU was used for immunization, suggesting that the Env TM ISD is not the only part of Env involved in mediating immunosuppression (16). Importantly, suppression was observed not only in co-immunizations but also when the Env vector was applied 3 weeks or even 3 months before immunization with a GagL_85-93_ epitope vaccine. Interestingly, we did not find an increased frequency of Tregs after Env immunization but showed that an appreciable frequency of Env-specific CD4^+^ T cells produced IL-10 (16). The suppression of CD8^+^ T-cell induction in immunization by Env therefore seems to be independent of the Treg induction observed in infection.

Our previous data suggested that Env SU also has immunosuppressive potential that is mainly exerted by changes in the cytokine milieu. In the present study, we aimed to analyze the immunosuppressive effect of retroviral Env proteins in immunization in more detail and to address the question of whether the immunosuppressive property of Env SU is a conserved feature shared by other retroviruses.

## Materials and methods

### Cells

A murine fibroblast cell line from *Mus dunni* (17), the murine lymphoma cell line FBL-3 (18), and the ovalbumin-expressing murine melanoma cell line B16-ova (19) were cultivated in RPMI medium (Invitrogen/Gibco, Karlsruhe, Germany). The human embryonic kidney cell line 293T (ATCC CRL-11268, LGC Standards, Wesel, Germany) was cultivated in a DMEM medium (Invitrogen/Gibco, Karlsruhe, Germany). All media were supplemented with 10% heat-inactivated fetal bovine serum (Invitrogen/Gibco), 20 µg/ml of ciprofloxacin (Fresenius Kabi AG, Bad Homburg vor der Höhe, Germany), and 50 μg/ml of gentamicin (AppliChem, Darmstadt, Germany), and cells were grown in a humidified atmosphere of 5% CO_2_ at 37°C.

### Plasmids

The F-MuLV-derived Leader-Gag encoding expression plasmid pCG.Leader-Gag and the F-MuLV-Env encoding plasmid pCG.env have been described before (20); both plasmids contain the respective whole open reading frames derived from F-MuLV strain FB29 under the control of a CMV-IE promoter. For the construction of Env SU-encoding plasmids, the genes encoding codon-optimized SU Env proteins of F-MuLV, Moloney-MuLV (Mo-MuLV), CasBr-MuLV, Hortulanus-MuLV (Hor-MuLV), 4070A-MuLV, feline leukemia virus (FeLV), avian leukosis/sarcoma virus (ALSV), and feline immunodeficiency virus (FIV) were synthesized by GeneArt (Thermo Fisher, Waltham, MA, USA) and cloned into the expression vector pcDNA that was modified with a β-globin intron cloned in front of and an OLLAS tag (21) at the end of the transgene cloning site (pcDNA_intron_OLLAS). Sequences encoding the codon-optimized SU Env protein of simian immunodeficiency virus (SIV) and human immunodeficiency virus (HIV) were amplified by PCR from plasmids described previously (22, 23) and cloned into pcDNA_intron_OLLAS. The pJI-based expression plasmid encoding hepatitis B virus surface antigen (HBsAg) has been described previously (24). The HIV-1 Gag expression plasmid pGag-eGFP encoding a Rev-independent full-length *gag* gene (p55M1234) has been described previously (25). Plasmids based on pVax encoding the influenza virus H1N1 A/Puerto Rico/8/1934 hemagglutinin (pV.HA) or nucleoprotein (pV.NP) have been described previously (26).

The purification of plasmids was performed by cesium chloride gradient centrifugation to obtain plasmid preparations with low endotoxin concentrations suitable for *in-vivo* use. The endotoxin concentrations of the DNA preparations were analyzed using the ToxinSensor endotoxin assay kit (GenScript, Piscataway, NJ, USA) and found to be below 0.002 endotoxin units (EU) per vaccine dose (range: 0.0002–0.0017 EU/dose). The expression of all SU Env proteins was verified by transfection of HEK 293T cells followed by Western blotting using the OLLAS tag for detection.

### Mice

Female CB6F1 (BALB/cAnNCrl x C57BL/6NCrl), C57BL/6NCrl, BALB/c, CBA, and C3H mice were acquired from Charles River Laboratories (Sulzfeld, Germany).

Mice of BALB/c background lacking expression of IL-10 in CD4^+^ cells (CD4-IL-10ko) were bred from Tg(CD4-cre) mice expressing cre recombinase under the control of CD4 enhancer, promoter, and silencer sequences (27) and IL-10tm1Roer mice carrying an insertion of loxP sites in the promoter region and downstream of exon 1 of IL-10 (kindly provided by Axel Roers, Heidelberg) (28). Mice of BALB/c background lacking expression of IL-10 in CD11c^+^ cells (CD11c-IL-10ko) were bred from Tg(Itax-cre)1-1Reiz/J mice expressing cre recombinase under the control of the integrin alpha X promotor/enhancer (29) and IL-10tm1Roer. BALB/c-based IL-10tm1Flv (IL-10-eGFP) mice express eGFP under the control of the IL-10 locus due to the insertion of an internal ribosomal entry site and the eGFP encoding sequences downstream of the IL-10 stop codon (30). CB6F1-IL-10-eGFP mice were bred by crossing IL-10-eGFP mice with C57BL/6NCrl mice. CD4-IL-10ko, CD11c-IL-10ko, and IL-10-IRES-eGFP mice were bred in the Animal Laboratory of the Institute for Virology at the University Hospital Essen.

Mice were housed in the animal facility of the Institute for Virology at the University Hospital Essen and treated in accordance with national law and the institutional guidelines of the University Hospital Essen, Essen, Germany. The study was approved by the North Rhine-Westphalia State Office for Nature, Environment and Consumer Protection (LANUV NRW). At the beginning of the experiments, all mice were between 7 and 9 weeks old and were housed in our in-house animal facility.

### DNA-based immunization

For DNA-based immunization, mice were anesthetized with 100 µg/g body weight of ketamine and 10 µg/g of xylazine by intraperitoneal application and injected intramuscularly with 25 µg of plasmid DNA in 30 µl of PBS into the *musculus gastrocnemius*. *In-vivo* electroporation was performed immediately after DNA injection with the BTX AgilePulse system using a 2 × 4 5-mm needle array (BTX Molecular Delivery Systems, Holliston, MA, USA) which was inserted into the muscle tissue at the site of DNA injection, applying two pulses of 450 V for 50 µs in a 1-ms interval, followed by 8 pulses of 110 V for 10 ms in 20-ms intervals. When animals were immunized twice, vaccinations were given in a 3-week interval.

### Tumor cell inoculation

A total of 5 × 10^6^ FBL-3 or B16-ova cells were inoculated subcutaneously into the *regio lumbalis* of CB6F1 mice. Tumor size was measured every second day using a Vernier caliper.

### Anti-IL-10 treatment

Mice were treated with an IL-10 neutralizing antibody (Jas5-2A5, BioXCell, Lebanon, NH, USA) by intraperitoneal injection of 100 µg of antibody in 100 µl of PBS three times per week, starting on the day of Env immunization. Anti-IL-10 antibody treatment was performed until day 13 after tumor cell injection in the tumor experiments or until 2 weeks after boost immunization in the co-immunization experiments.

### FV and challenge infection

An uncloned, lactate dehydrogenase-elevating virus (LDV)-free FV stock was obtained from BALB/c mouse spleen cell homogenate (10%, wt/vol) 14 days after infection with a B-tropic, polycythemia-inducing FV complex (31). CB6F1 mice were infected with 5,000 spleen focus-forming units (SFFU) diluted in 100 µl of PBS by intravenous injection into the *vena caudalis mediana*.

### Infectious center assay

Twenty-one days post-challenge (p.c.), animals were sacrificed by cervical dislocation, the spleens were removed and weighed, and single-cell suspensions were prepared. Serial dilutions of isolated spleen cells were seeded onto *M*. *dunni* cells, and cells were incubated under standard tissue culture conditions for 3 days. When cells reached ~100% confluence, they were fixed with ethanol and labeled with F-MuLV Env-specific monoclonal antibody MAb 720 (32) and then with a horseradish peroxidase (HRP)-conjugated rabbit anti-mouse Ig antibody (Dako, Hamburg, Germany). The assay was developed using aminoethylcarbazole (Sigma-Aldrich, Deisenhofen, Germany) as substrate to detect foci. The resulting foci were counted, and infectious centers (IC)/10^8^ spleen cells were calculated.

### Flow cytometric analysis of eGFP expression

For the analysis of eGFP expressing cells from IL-10-eGFP reporter mice, draining (popliteal, inguinal) and non-draining lymph nodes (axillary, brachial, cervical) were isolated, and single-cell suspensions were stained with PerCP5.5-anti-CD25, AF647-anti-CD62L, BUV395-anti-CD4, BUV563-anti-CD43 (all from Becton-Dickinson, Franklin Lakes, NJ, USA), AF700-anti-CD3 (Invitrogen, Waltham, MA, USA), BV711-anti-CD44, PE-Dazzle594-anti-CD11c, BV605-anti-CD11b (BioLegend, San Diego, CA, USA), eFluor450-anti-Gr1, and Fixable Viability Dye eFluor 780 (eBioscience, Frankfurt, Germany).

Data were acquired on a BD FACSymphony A5 flow cytometer (Becton-Dickinson) and analyzed using FlowJo software (TreeStar, Ashland, OR, USA). Exemplary plots showing the gating strategy are shown in **Supplementary Figure 2**.

### Tetramer and dextramer staining of transgene-specific T cells

For H2-K^b^ Ova_257-264_ (SIINFEKL)-specific CD8^+^ T-cell analysis, spleen cells were obtained 12 days after tumor inoculation and stained with AF647-coupled dextramer loaded with Ova_257-264_ peptide (Dex^OT–I^; Immudex, Copenhagen, Denmark), FITC-anti-CD11b, PerCP-anti-CD43, BV421-anti-CD8 (all from BioLegend, San Diego, CA, USA), Fixable Viability Dye eFluor 780, PE-Cy7-anti-CD62L (eBioscience), and BV510-anti-CD44 (Becton-Dickinson).

The frequency of CD8^+^ T cells specific for the H2-D^b^-restricted Leader-Gag-derived epitope GagL_85–93_ [CCLCLTVFL (33)] was analyzed 14 days after DNA-based immunization in peripheral blood cells after erythrocyte lysis or in spleen cells after tumor cell inoculation. Cells were stained with PE-coupled MHC I tetramer [TetI^GagL^; carrying the peptide AbuAbuLAbuLTVFL, in which cysteine residues of the original GagL_85-93_ amino acid sequence were replaced by aminobutyric acid (Abu) to prevent disulfide bonding; MBL, Woburn, MA, USA], PerCP-anti-CD43, BV421-anti-CD8, BV510-anti-CD44, PE-Cy7-anti-CD62L (all from BioLegend), and Fixable Viability Dye eFluor 780 (eBioscience).

Data were acquired on a BD FACSymphony A5 flow cytometer (Becton-Dickinson) and analyzed using FlowJo software (TreeStar). Exemplary plots showing the gating strategy are shown in **Supplementary Figure 2**.

### Peptide stimulation and intracellular cytokine staining

For the analysis of cytokine production by antigen-specific CD8^+^ T cells, splenocytes or blood cells after erythrocyte lysis were stimulated for 6 h *in vitro* with 5 µg/ml of peptide as indicated below in the presence of 2 µg/ml of brefeldin A in RPMI medium supplemented with 10% FCS, 2 mM of L-glutamine, 20 mM of HEPES, 50 µM of β-mercaptoethanol, and 50 µg/ml of gentamicin.

For the analysis of ova-specific CD8^+^ T cells, cells were stimulated with the peptide Ova_257-264_ (SIINFEKL; H2-K^b^-restricted). For the analysis of F-MuLV Leader-Gag-specific CD8^+^ T cells, cells were stimulated with the peptide GagL_85-93_ (AbuAbuLAbuLTVFL; cysteines of the original sequence CCLCLTVFL were replaced by Abu). For the analysis of HIV Gag-specific CD8^+^ T cells, cells were stimulated with the peptides Gag_189-203_ (NTVGGHQAAMQMLKE) and Gag_193-207_ (GHQAAMQMLKETINE) containing the H2-K^d^-restricted Gag_192-200_ epitope (GGHQAAMYM), with the peptides Gag_73-87_ (EELRSLYNTVATLYC) and Gag_77-91_ (SLYNTVATLYCVHQR) containing the H2-K^d^-restricted epitope Gag_78-85_ (LYNTVATL), and with the peptides Gag_273-287_ (IVRMYSPTSILDIRQ) and Gag_269-283_ (GLNKIVRMYSPTSIL) containing the H2-K^d^-restricted epitope Gag_274-282_ (VRMYSPTSI). For the analysis of HBsAg-specific CD8^+^ T cells, cells were stimulated with the peptide HBsAg_190-197_ (VWLSVIWM; H2-K^b^-restricted). For the analysis of influenza-specific CD8^+^ T cells, cells were stimulated with HA_462-470_ (LYEKVKSQL; H2-K^d^-restricted), HA_518-526_ (IYSTVASSL; H2-K^d^-restricted), HA3_54-362_ (IEGGWTGMI; H2-K^k^-restricted), and HA_259-266_ (FEANGNLI; H2-K^k^-restricted), or NP366-374 (ASNENMETM; H2-D^b^-restricted), NP147-155 (TYQRTRALV; H2-D^b^-restricted), and NP50-57 (SDYEGRLI; H2-K^k^-restricted). HIV Gag peptides were obtained from the NIH AIDS Reagent Program; all the other peptides were obtained from Peptides&Elephants, Berlin, Germany.

All stimulations were stained with FITC-anti-IFNy, APC-anti-GzmB (Molecular Probes, ThermoFisher Scientific, Waltham, MA, USA), PerCP-anti-CD43, BV421-anti-CD8 (all from BioLegend), BV510-anti-CD44 (Becton-Dickinson), and Fixable Viability Dye eFluor 780 (eBioscience). Before intracellular staining, cells were fixed with PBS containing 2% paraformaldehyde and permeabilized with 0.1% saponin buffer.

Data were acquired on a BD FACSymphony™ A5 flow cytometer (Becton-Dickinson) and analyzed using FlowJo software (TreeStar). Exemplary plots showing the gating strategy are shown in **Supplementary Figure 2.**

### Statistical analysis

Statistical analyses were performed in GraphPad Prism 8 software (GraphPad Software, La Jolla, CA, USA). For the comparison of multiple groups, one-way analysis of variance on ranks with Dunn’s post-test was performed. For the comparison of two groups, the Mann–Whitney rank sum test was used. Numerical results for *p*-values are listed in the **Supplementary Tables.**

## Results

### Induction of IL-10 by Env (SU+TM) immunization in CD4^+^ T cells

**Figure 1:**
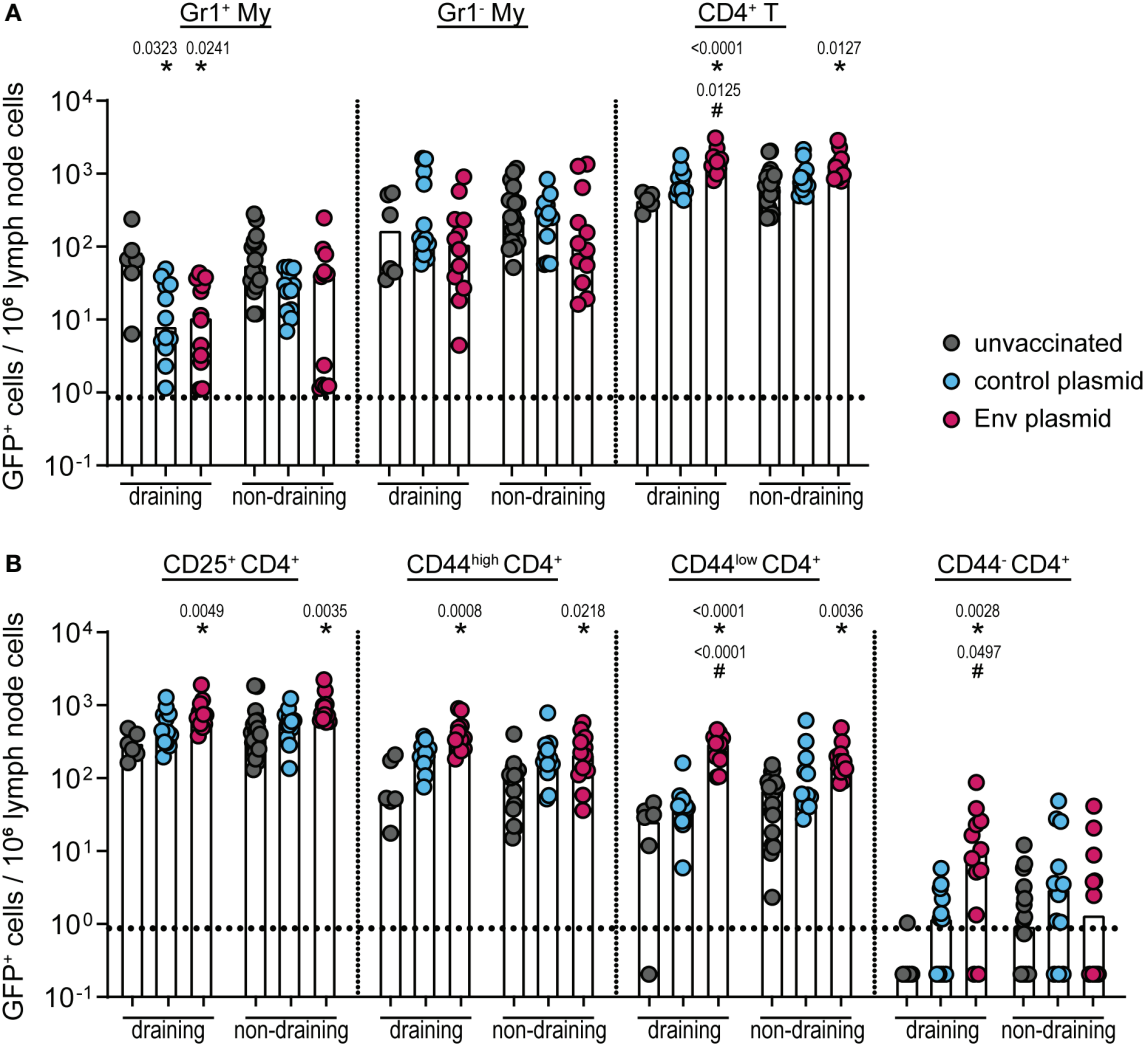
IL-10 expression by different cell types after pCG.env immunization of CB6F1-IL-10-eGFP reporter mice. CB6F1-IL-10-eGFP mice were immunized once with 25 µg of F-MuLV Env SU+TM encoding plasmid pCG.env by intramuscular injection followed by *in-vivo* electroporation. Two weeks after the immunization, mice were sacrificed and draining and non-draining lymph nodes were isolated for flow cytometric analysis of myeloid cells and CD4^+^ T cells. The frequency of eGFP^+^ cells of the indicated cell types in 10^6^ lymph node cells is shown for Gr1^+^ and Gr1^−^ CD11b^+^ myeloid cells and CD4^+^ T cells **(A)** and for the indicated CD4^+^ T-cell subsets **(B)**. Data from 12 mice per group were acquired in two independent experiments. Each dot indicates an individual mouse, and bars indicate median values. Statistically significant differences compared with the frequency of the respective cell population in unvaccinated mice are indicated by *, and differences compared with control plasmid-immunized mice are indicated by ^#^ (*p* < 0.05, Kruskal–Wallis one-way ANOVA on ranks, Dunn’s post-test).

We previously observed that the immunization with either the whole F-MuLV Env (SU+TM) or F-MuLV Env SU alone suppresses the simultaneous or subsequent induction of CD8^+^ T-cell responses, which might be mechanistically linked to an increased frequency of IL-10-producing CD4^+^ T cells after Env immunization (16). Our previous experiments did not aim to yield information on whether the induction of IL-10-producing CD4^+^ T cells is directly stimulated by Env contact or mediated by IL-10-producing myeloid cells. Thus, to characterize the cells that contribute to the IL-10 production after Env (SU+TM) immunization in more detail, we performed DNA plasmid immunization of CB6F1-IL-10-eGFP reporter mice (vaccination scheme in **Supplementary Figure 1A**). Two weeks after a single immunization with the F-MuLV Env (SU+TM) encoding plasmid pCG.env, draining and non-draining lymph nodes were isolated and analyzed for eGFP expression in myeloid cells (CD3^−^ CD11b^+^) and CD4^+^ T cells (CD3^+^ CD4^+^ CD11b^−^; **Figure 1**). In the myeloid cell compartment, we did not observe any increased frequencies of eGFP^+^ cells in Env-immunized mice compared with unvaccinated or control plasmid-immunized mice (**Figure 1A**). In contrast, the frequency of eGFP^+^ CD4^+^ T cells was significantly increased both in draining and non-draining lymph nodes of Env-immunized mice compared with unvaccinated mice and also compared with control plasmid-immunized mice in draining lymph nodes. A more detailed analysis of the CD4^+^ T-cell phenotype showed that not only did the antigen-experienced CD25^+^ or CD44^high^ CD4^+^ T-cell subsets contribute to the expanded eGFP^+^ CD4^+^ T-cell compartment in Env immunized mice, but also CD44^low^ and CD44^−^ CD4^+^ T cells were found to have an increased frequency of eGFP^+^ cells in Env-immunized compared with unvaccinated mice and control plasmid-immunized mice (**Figure 1B**). These findings suggest that there is no significant contribution of IL-10-expressing myeloid cells after Env immunization, and imply CD4^+^ T cells as the main contributors to the IL-10 production after Env immunization.

### Suppression of HIV Gag-specific CD8^+^ T-cell responses by F-MuLV Env (SU+TM) in BALB/c and IL-10ko mice

**Figure 2:**
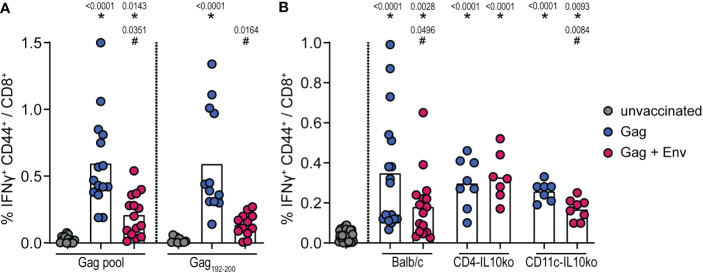
Suppression of HIV Gag-specific CD8^+^ T-cell responses by F-MuLV Env. CB6F1 **(A)** and BALB/c, CD4-IL-10ko, and CD11c-IL-10ko mice **(B)** were immunized once with 25 µg of the plasmid pGag-eGFP encoding HIV Gag, or they were co-immunized once with pGag-eGFP and the plasmid pCG.env encoding F-MuLV Env SU+TM (25 µg each) by intramuscular injection followed by *in-vivo* electroporation. Two weeks after the immunization, mice were sacrificed and spleens were isolated, and the frequency of Gag-specific IFNy^+^ CD44^+^ CD8^+^ cells was analyzed by flow cytometry after *in-vitro* restimulation as indicated **(A)** or with an HIV Gag peptide pool **(B)**. Data from 15 **(A)** and 8/7 **(B)** mice per group were acquired in three **(A)** or two **(B)** independent experiments. Each dot indicates an individual mouse, and bars indicate mean values. Statistically significant differences compared with the unvaccinated group are indicated by *, and differences compared with the HIV Gag group are indicated by ^#^ (*p* < 0.05, Kruskal–Wallis one-way ANOVA on ranks, Dunn’s post-test).

We previously observed the suppression of CD8^+^ T-cell responses by F-MuLV Env for both F-MuLV Leader-Gag- and ovalbumin-specific CD8^+^ T-cell responses (16). We now analyzed whether the same suppressive effect would also be observed for CD8^+^ T-cell responses against HIV Gag (vaccination scheme in **Supplementary Figure 1B**). When we immunized CB6F1 mice with an HIV Gag encoding plasmid with or without the co-immunization with an F-MuLV Env (SU+TM) plasmid and analyzed the induction of HIV Gag-specific CD8^+^ T cells, we found a robust induction of CD8^+^ T cells in Gag-immunized mice, which was significantly reduced when mice were co-immunized with the F-MuLV Env (SU+TM) plasmid (**Figure 2A**). Since the analyzed CD8^+^ T cells were specific for HIV Gag epitopes restricted by H2-K^d^, we were able to perform the same experiment in BALB/c mice and in BALB/c-based mice with inactivation of IL-10 in CD4^+^ or CD11c^+^ cells, allowing us to directly analyze the contribution of CD4^+^ and CD11c^+^ cell-derived IL-10 to the Env-mediated suppression. Importantly, we found the same suppressive effect of Env co-immunization on HIV Gag-specific CD8^+^ T-cell responses in BALB/c mice as we had seen before in CB6F1 mice (**Figure 2B**). Interestingly, the suppressive effect was alleviated in CD4-IL-10ko mice, where we found no difference in HIV Gag-specific CD8^+^ T-cell responses after Env co-immunization compared with HIV Gag immunization alone. On the other hand, the suppressive effect in CD11c-IL-10ko mice was similar to that observed in BALB/c mice, corroborating our findings from the IL-10-eGFP reporter mice that IL-10-producing CD4^+^ T cells contribute more to the Env-specific IL-10 response than myeloid cells and establishing the mechanistic role of IL-10-producing CD4^+^ T cells as mediators of the Env-induced suppression of CD8^+^ T-cell responses.

### Suppression of CD8^+^ T-cell responses against different immunogens

**Figure 3:**
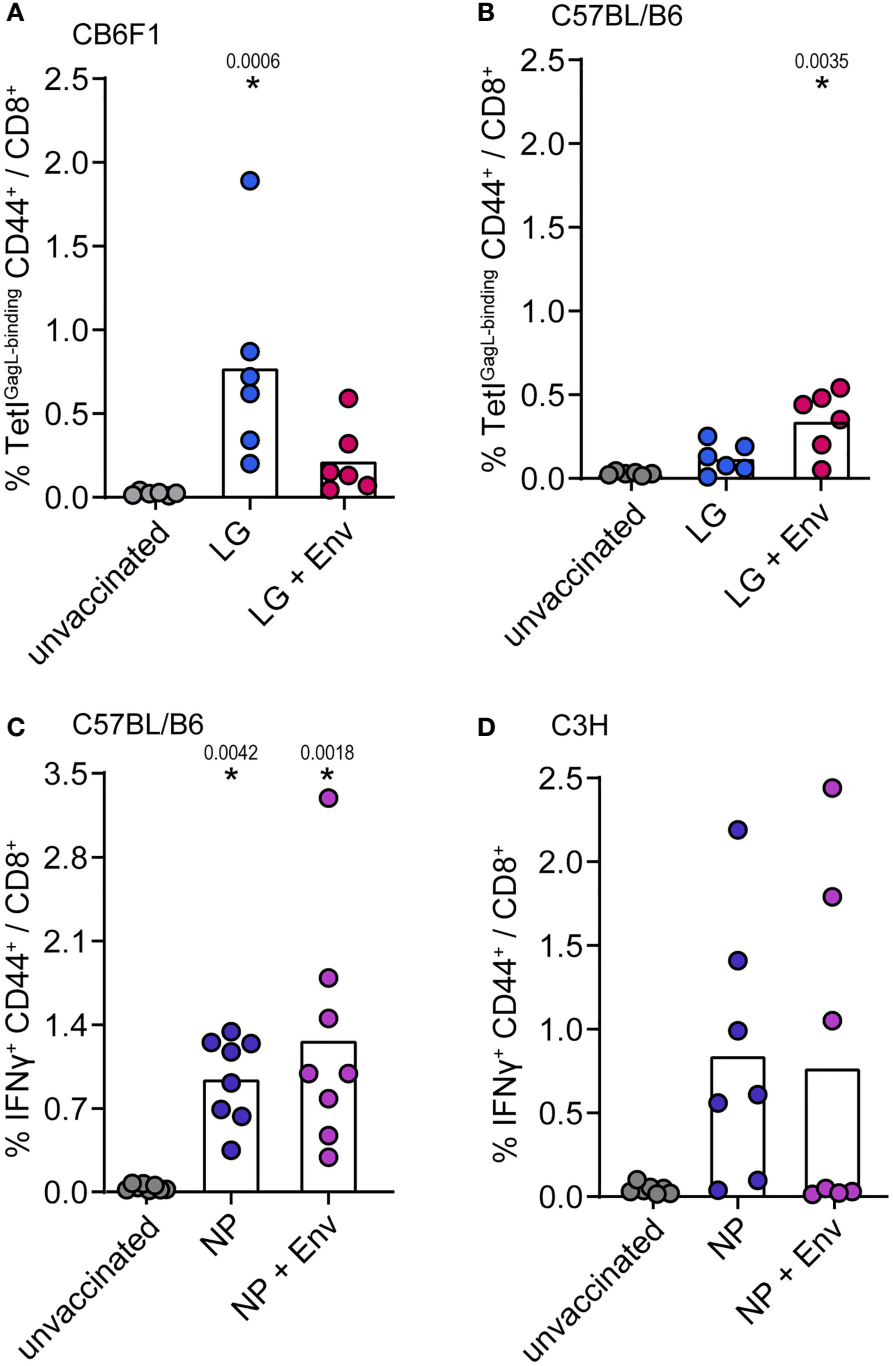
No suppression of influenza nucleoprotein-specific CD8^+^ T-cell responses. CB6F1 **(A)** and C57BL/B6 **(B)** mice were immunized once with 25 µg of the F-MuLV Leader-Gag encoding plasmid pCG.Leader-Gag alone (LG) or in combination with the F-MuLV Env SU+TM encoding plasmid pCG.env (LG + Env). **(C)** C57BL/6 mice and C3H mice **(D)** were immunized once with 25 µg of the nucleoprotein (NP) encoding plasmid pV.NP alone (NP) or in combination with pCG.env (NP + Env). Vaccinations were performed by intramuscular injection followed by *in-vivo* electroporation. Two weeks after immunization, mice were sacrificed and spleens were isolated, and the frequency of immunogen-specific CD8^+^ T cells was determined by MHC-I tetramer staining **(A, B)** or by intracellular cytokine staining after *in-vitro* restimulation with the appropriate peptides **(C, D)**. Data from 8 **(C)**, 7 **(D)**, and 6 **(A, B)** mice per group were acquired in two independent experiments. Each dot indicates an individual mouse, and bars indicate mean values. Statistically significant differences compared with the unvaccinated group are indicated by * (*p* < 0.05, Kruskal–Wallis one-way ANOVA on ranks, Dunn’s post-test).

We next wanted to analyze whether the suppressive effect of Env (SU+TM) on CD8^+^ T-cell responses could also be observed in other mouse strains and for other immunogens. In confirmation of our previous findings (15), the immunization of CB6F1 mice with pCG.Leader-Gag resulted in the induction of a potent GagL_85-93_-specific CD8^+^ T-cell response, which was impaired when mice were co-immunized with pCG.Env (**Figure 3A**; vaccination scheme in **Supplementary Figure 1C**). Surprisingly, this effect was not observed in C57BL/6 mice, as mice co-immunized with Leader-Gag and Env encoding plasmids had higher GagL_85-93_-specific CD8^+^ T-cell responses than mice immunized with Leader-Gag plasmid alone (**Figure 3B**). Also for some other immunogens, we did not observe suppression of CD8^+^ T-cell responses: influenza virus nucleoprotein-specific CD8^+^ T-cell responses were not impaired in C57BL/6 or C3H mice (**Figures 3C, D**) nor were hepatitis B virus surface antigen (HBsAg)-specific CD8^+^ T-cell responses in CB6F1 mice and influenza virus hemagglutinin-specific CD8^+^ T-cell responses in BALB/c, C3H, or CBA mice when the F-MuLV Env plasmid was co-administered with the respective immunogen plasmids (**Supplementary Figure 1**). These results show that the suppression of CD8^+^ T cells depends on both the immunogen and the mouse strain, suggesting that some host factors may be involved.

### Suppression of tumor control by F-MuLV Env (SU+TM) immunization

**Figure 4:**
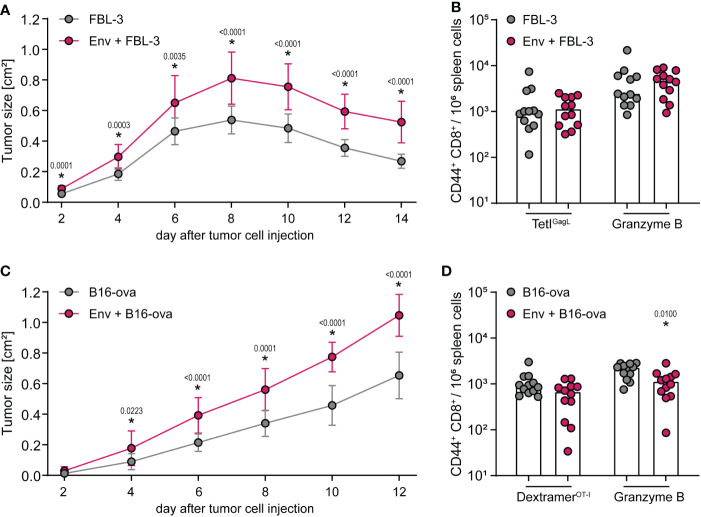
Control of tumor cell growth is impaired after F-MuLV Env immunization. **(A, B)** CB6F1 mice were inoculated with 5 × 10^6^ FBL-3 cells by subcutaneous injection with or without an immunization with 25 µg of F-MuLV Env (SU+TM) encoding plasmid pCG.env 3 days before. Tumor size was measured every other day **(A)**, and 14 days after tumor cell injection, mice were sacrificed and the frequency of GagL_85-93_-specific CD8^+^ T cells and of granzyme B^+^ CD8^+^ T cells in spleen cells was determined by flow cytometry **(B)**. **(C, D)** CB6F1 mice were inoculated with 5 × 10^6^ B16-ova cells by subcutaneous injection with or without an immunization with 25 µg of F-MuLV Env encoding plasmid pCG.env 3 days before. Tumor size was measured every other day **(C)**, and 12 days after tumor cell injection, mice were sacrificed and the frequency of Ova_257-264_-specific CD8^+^ T cells and of granzyme B^+^ CD8^+^ T cells in spleen cells was determined by flow cytometry **(D)**. Data from 12 mice per group were acquired in three independent experiments. **(A, C)** The mean tumor size with standard deviation. **(B, D)** Each dot indicates an individual mouse, and bars indicate median values. Statistically significant differences compared with the unvaccinated group at the same time point **(A, C)** or compared with the respective cells of unvaccinated mice **(B, D)** are indicated by * (*p* < 0.05, Mann–Whitney rank sum test).

We have shown before that the suppression of CD8^+^ T-cell responses occurs when vector or DNA vaccines encoding F-MuLV Env (SU+TM) and the CD8^+^ T-cell immunogen are combined or administered sequentially. We next wanted to analyze the influence of an F-MuLV Env (SU+TM) immunization on the CD8^+^ T-cell response to subsequent tumor cell inoculation. We therefore performed experiments using the F-MuLV-induced lymphoma cell line FBL-3 and the ovalbumin-expressing B16 melanoma cell line in CB6F1 mice. Of note, the FBL-3 cell line expresses F-MuLV antigens including Env (34), whereas B16-ova are F-MuLV-unrelated. In the tumor experiments, the mice were immunized with the F-MuLV Env SU+TM plasmid 3 days before tumor cell inoculation (**Supplementary Figure 1D**), to ensure the presence of Env at the time point of tumor cell inoculation, i.e., when tumor antigens were introduced and presented to the immune system. When mice were inoculated with FBL-3 cells, tumor growth was significantly increased when mice had received an F-MuLV Env immunization 3 days before tumor cell injection in comparison with non-vaccinated mice (**Figure 4A**), even though both groups of mice were able to control the tumor growth. When we analyzed the frequency of GagL_85-93_-specific CD8^+^ T cells and of CD8^+^ T cells producing the cytotoxic effector molecule granzyme B 14 days after tumor cell inoculation, we did not find any significant differences, which may be due to the fact that the tumors were already largely cleared at that time point (**Figure 4B**). When mice were inoculated with B16-ova tumor cells, we similarly found that mice developed significantly larger tumors when they had received an F-MuLV Env immunization 3 days before tumor cell inoculation than non-vaccinated mice, but in this tumor model, none of the mice were able to clear the tumor (**Figure 4C**). When we analyzed the frequency and effector function of Ova_257-264_-specific CD8^+^ T cells, we found that Env-immunized mice indeed showed a tendency toward fewer Ova_257-264_-specific CD8^+^ T cells and had significantly fewer granzyme B-producing CD8^+^ T cells (**Figure 4D**). Overall, these experiments show that the Env-mediated immune suppression also leads to reduced control of a subsequently applied tumor, even if the tumor also expresses F-MuLV Env as in the case of the FBL-3 cells.

### Alleviation of tumor suppression by anti-IL-10 antibody treatment

**Figure 5:**
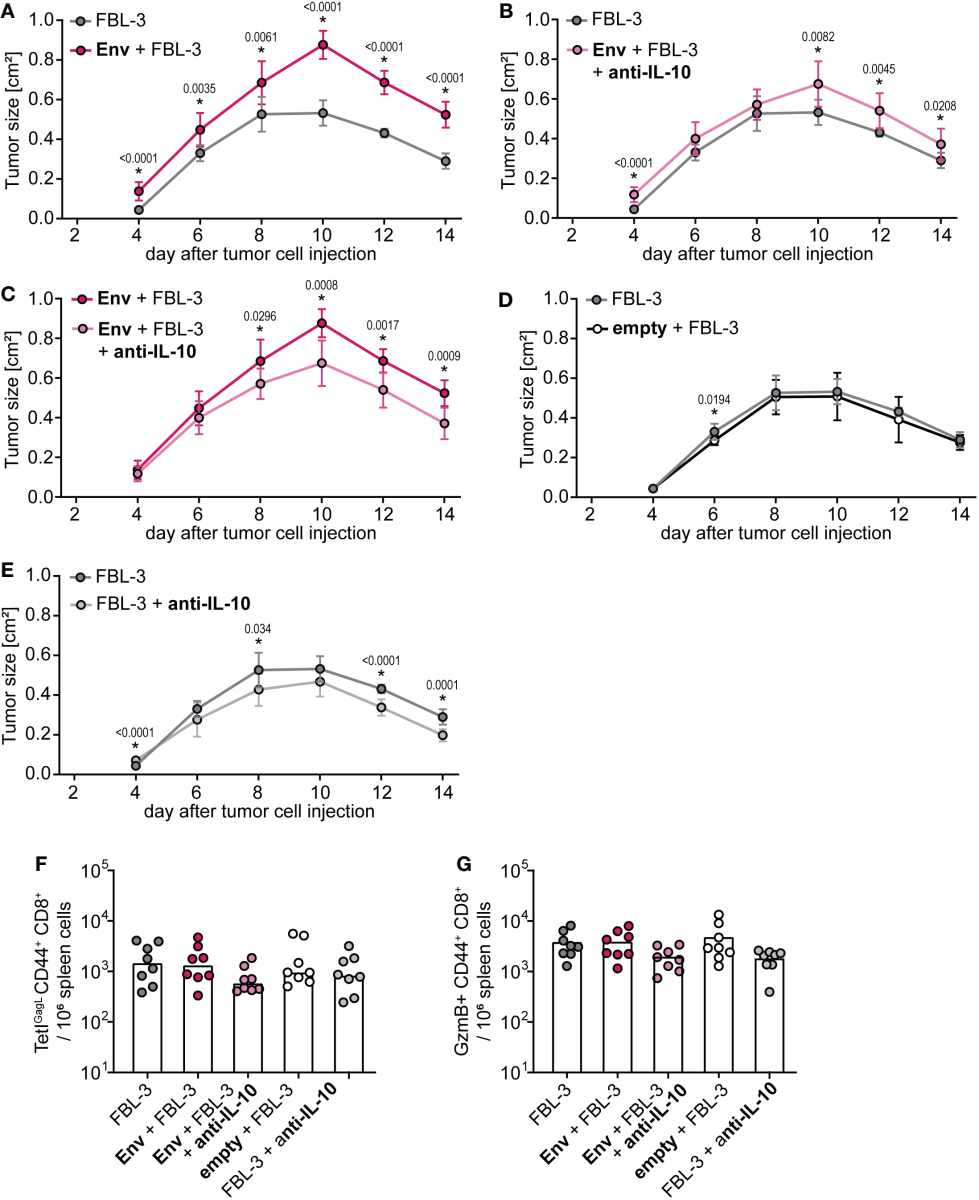
Control of tumor growth after F-MuLV Env immunization is restored by anti-IL-10 antibody treatment. **(A–E)** CB6F1 mice were inoculated with 5 × 10^6^ FBL-3 cells by subcutaneous injection with or without an immunization with 25 µg of F-MuLV Env encoding plasmid pCG.env or an empty control plasmid 3 days before. Mice were treated with an anti-IL-10 antibody three times per week or left untreated, and tumor size was measured every other day. **(F, G)** Fourteen days after tumor cell injection, mice were sacrificed and the frequency of GagL_85-93_-specific CD8^+^ T cells **(F)** and of granzyme B^+^ CD8^+^ T cells in spleen cells was determined by flow cytometry **(G).** Data from eight mice per group were acquired in one experiment. **(A–E)** The mean tumor size with standard deviation. **(F, G)** Each dot indicates an individual mouse, and bars indicate median values. Statistically significant differences between the groups shown in the individual plots **(A–E)** at the same time point are indicated by * [*p* < 0.05, Mann–Whitney rank sum test **(A–E)** or Kruskal–Wallis one-way ANOVA on ranks **(F, G)**].

To prove that the suppression of tumor control is also mediated by IL-10, we performed an FBL-3 tumor control experiment in which we treated mice with an IL-10 blocking antibody, starting on the day of F-MuLV Env (SU+TM) immunization (**Supplementary Figure 1E**). The control of tumor growth was again significantly impaired in mice that had received an Env immunization compared with unvaccinated mice (**Figure 5A**), and it was significantly improved when Env-immunized mice were treated with an anti-IL-10 antibody (**Figures 5B**, **C**), proving the mechanistic role of IL-10 also in the suppression of FBL-3 tumor control. Importantly, immunization with an empty plasmid did not lead to a significant change in tumor control (**Figure 5D**). While the effect was not very pronounced, we also observed slightly improved tumor control when unvaccinated mice were treated with the anti-IL-10 antibody (**Figure 5E**), which can be explained by the fact that the FBL-3 tumor cells also express Env and may therefore be expected to similarly induce IL-10 expression. As before, no significant differences in CD8^+^ T-cell responses were observed at the end of the experiment (**Figures 5F, G**), which is likely due to the time point of analysis, as mentioned above.

### Suppression of CD8^+^ T-cell responses by Env SU proteins

**Figure 6:**
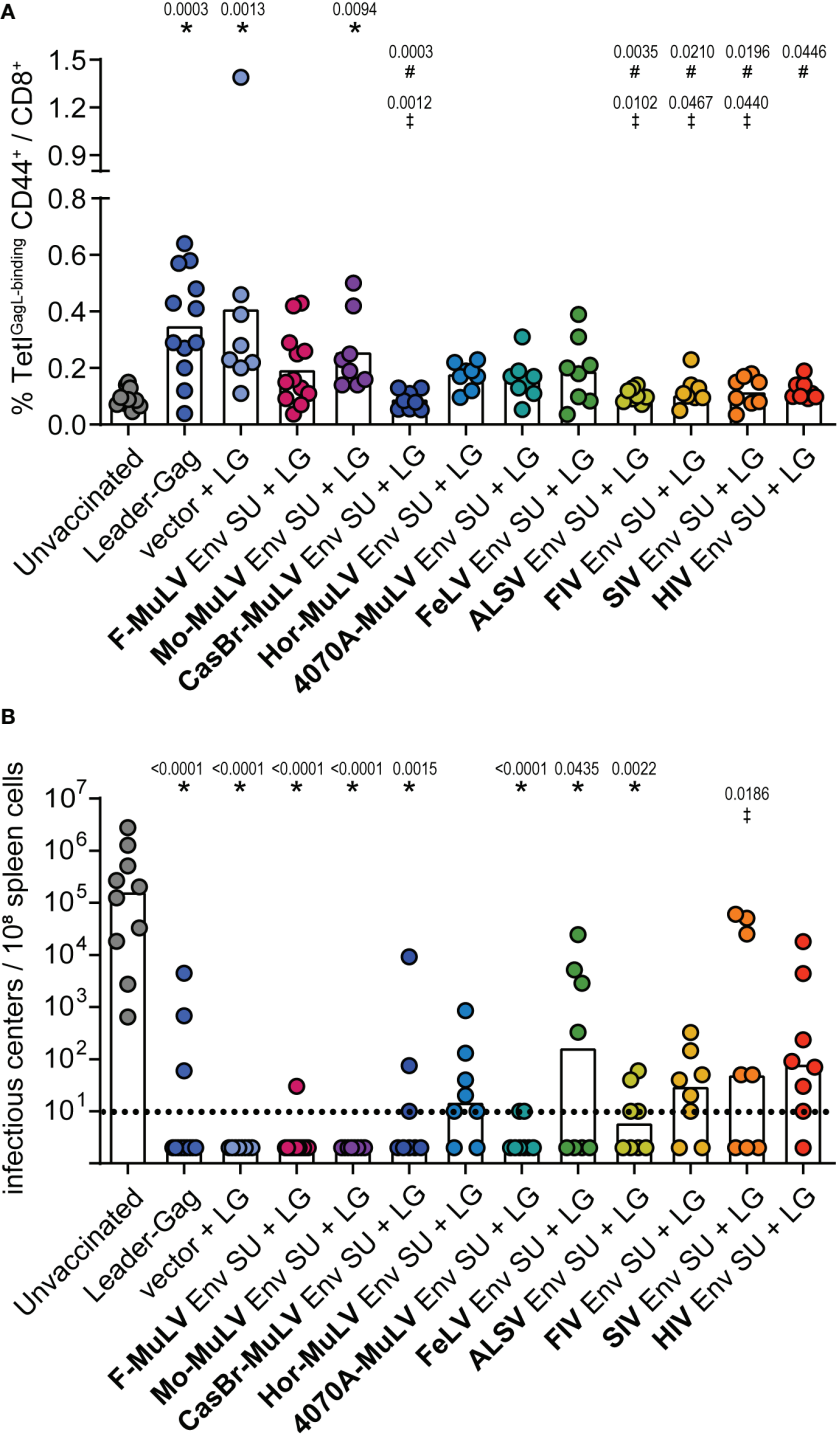
F-MuLV Leader-Gag-specific CD8^+^ T-cell response after co-immunization with different Env SU-encoding plasmids. CB6F1 mice were immunized twice in a 3-week interval with 25 µg of pCG.Leader-Gag with or without co-immunization with 25 µg of a plasmid encoding SU Env of F-MuLV, Mo-MuLV, CasBr-MuLV, Hor-MuLV, 4070A-MuLV, FeLV, ALSV, FIV, SIV, or HIV or a control plasmid by intramuscular injection followed by *in-vivo* electroporation. Two weeks after the boost immunization, the frequency of GagL_85-93_-specific CD8^+^ T cells was analyzed in peripheral blood **(A)**. Three weeks after booster vaccination, the mice were infected with 5,000 SFFU FV by intravenous injection. Three weeks after the FV challenge, mice were sacrificed and the viral loads in the spleens were determined **(B)**. Data from 8 to 12 mice per group were acquired in two to three independent experiments. Each dot indicates an individual mouse, and bars indicate mean **(A)** or median values **(B)**. Statistically significant differences compared with the unvaccinated group are indicated by *, differences compared with the Leader-Gag group are indicated by ^#^and differences compared with the vector + LG group are indicated by ^‡^ (*p* < 0.05, Kruskal–Wallis one-way ANOVA on ranks, Dunn’s post-test).

Having shown in various settings that F-MuLV Env (SU+TM) exerts suppressive effects on the induction of CD8^+^ T-cell responses and, importantly, that this is true also for the Env SU domain alone (16), it was intriguing to know if the suppression by Env SU alone is also a universal property of retrovirus Env proteins. We therefore performed co-immunization experiments in which CB6F1 mice were immunized twice either with Leader-Gag alone or in combination with plasmids encoding the Env SU domains of F-MuLV, Moloney-MuLV, CasBr-MuLV, Hortulanus-MuLV, 4070A-MuLV, FeLV, ALSV, FIV, SIV, or HIV (vaccination scheme in **Supplementary Figure 1F**). While most mice that received the Leader-Gag vaccine alone and also mice that received the Leader-Gag vaccine together with a control plasmid mounted a robust GagL_85-93_-specific CD8^+^ T-cell response, all mice that were co-immunized with an Env SU-encoding plasmid tended to have reduced GagL_85-93_-specific CD8^+^ T-cell responses, with the strongest reduction in mice co-immunized with Env SU of CasBr-MuLV, ALSV, FIV, SIV, and HIV compared with mice immunized with Leader-Gag alone or co-immunized with an empty control plasmid (**Figure 6A**). Consequently, not all mice were protected from a subsequent challenge infection with FV, and more than half of the mice co-immunized with Hortulanus-MuLV, FeLV, FIV, SIV, or HIV Env SU had detectable viral loads in the spleens 21 days after FV infection (**Figure 6B**).

### Suppression of CD8^+^ T cells by Env SU partially alleviated by anti-IL-10 antibody treatment

**Figure 7:**
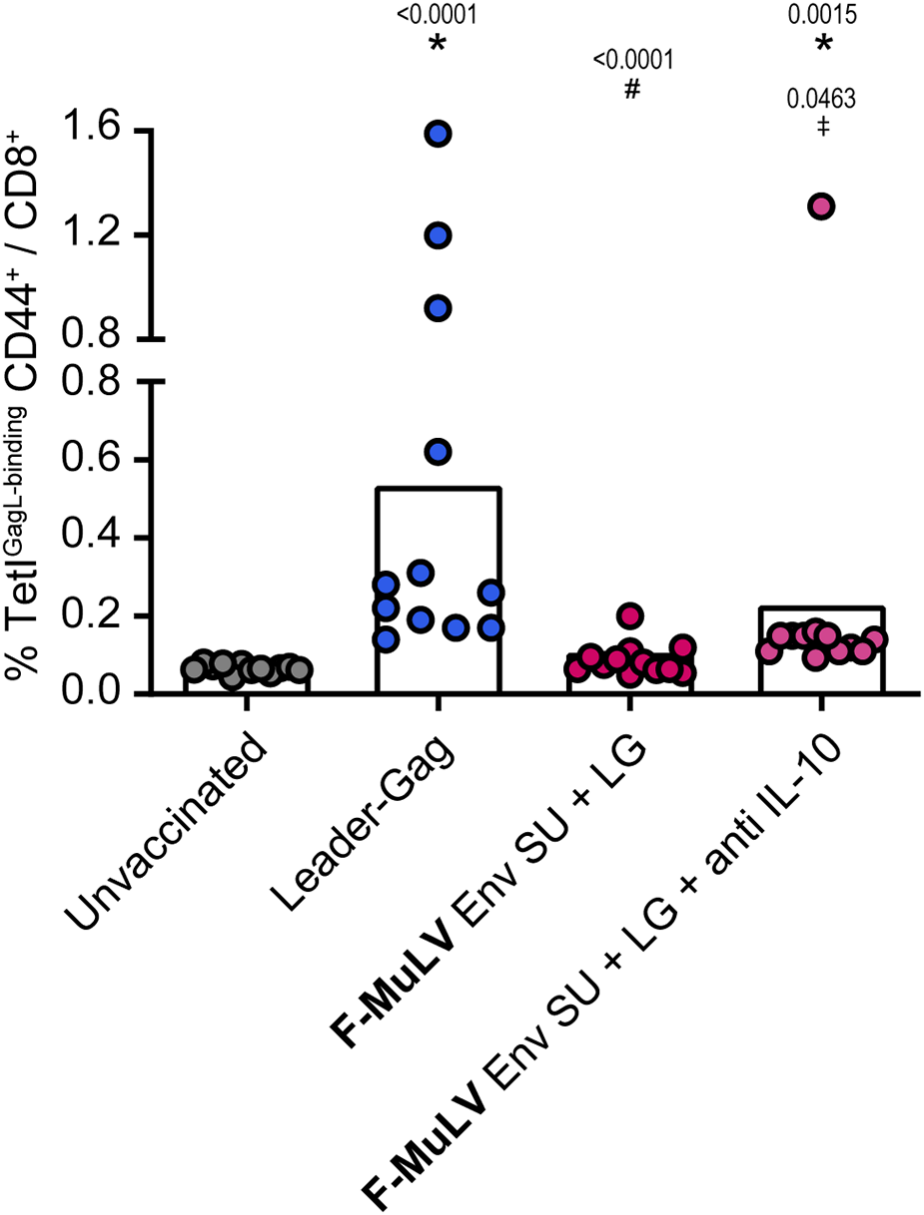
Inhibition of Leader-Gag-specific CD8^+^ T-cell response by F-MuLV Env SU is partially alleviated by anti-IL-10 antibody treatment. CB6F1 mice were immunized twice in a 3-week interval with 25 µg of pCG.Leader-Gag with or without co-immunization with 25 µg of a plasmid encoding F-MuLV Env SU by intramuscular injection followed by *in-vivo* electroporation. One group of mice received an anti-IL-10 treatment three times per week starting on the day of first immunization. Two weeks after the boost immunization, the frequency of GagL_85-93_-specific CD8^+^ T cells was analyzed in peripheral blood. Data from 12 mice per group were acquired in two independent experiments. Each dot indicates an individual mouse, and bars indicate mean values. Statistically significant differences compared with the unvaccinated group are indicated by *, differences compared with the Leader-Gag group are indicated by ^#^and differences to the F-MuLV Env SU + LG group are indicated by ^‡^ (*p* < 0.05, Kruskal–Wallis one-way ANOVA on ranks, Dunn’s post-test).

To analyze whether the suppressive effect of Env SU is also mediated by IL-10, we performed a co-immunization experiment in which we treated mice with an IL-10 blocking antibody. As before, Leader-Gag-immunized mice mounted a robust GagL_85-93_-specific CD8^+^ T-cell response that was severely impaired when mice were co-immunized with F-MuLV Env SU (**Figure 7**). Importantly, the GagL_85-93_-specific CD8^+^ T-cell response was significantly higher when co-immunized mice received anti-IL-10 treatment compared with co-immunization alone, indicating that F-MuLV Env SU-mediated suppression is at least partially mediated by IL-10.

### Suppression of CD8^+^ T cells by Env SU in sequential immunization

**Figure 8:**
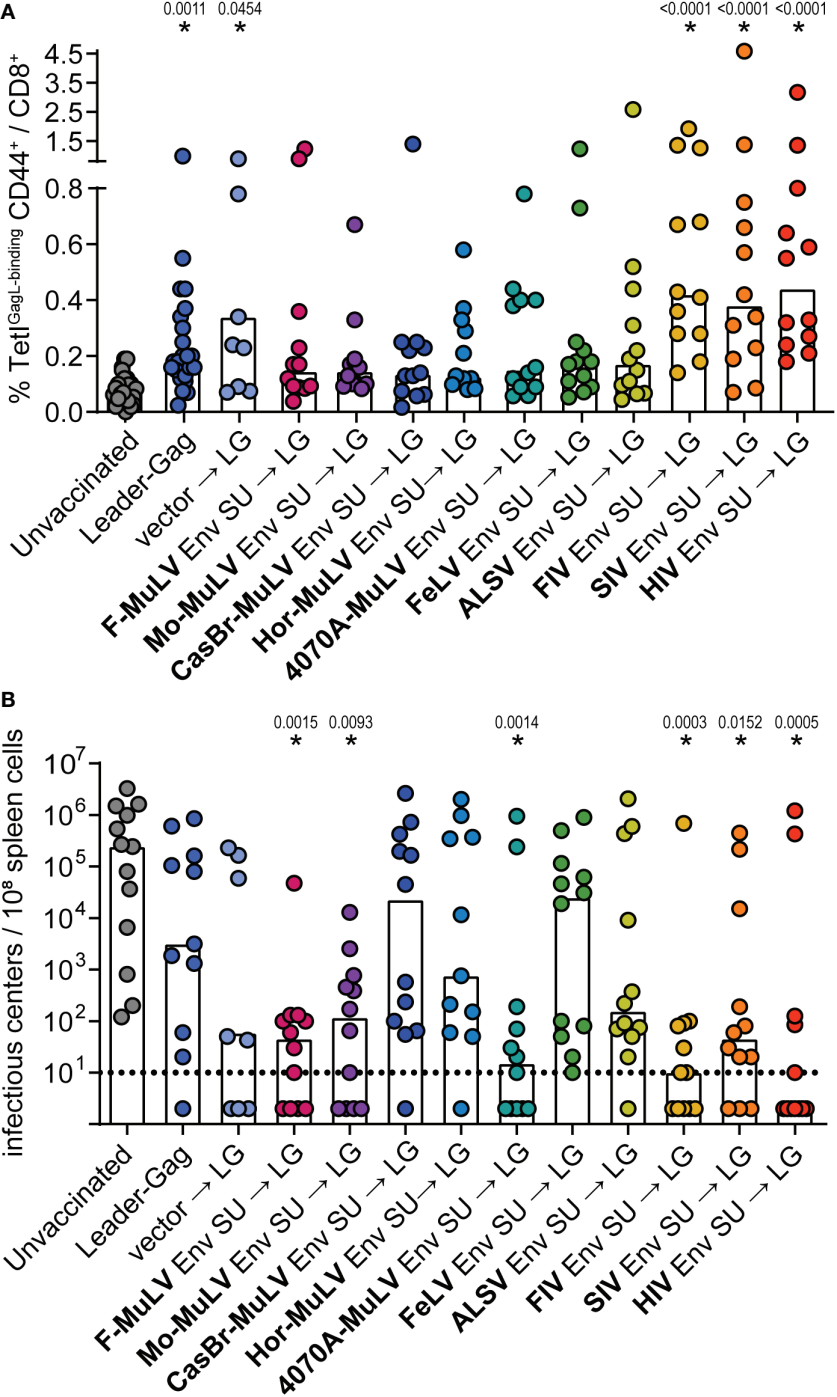
F-MuLV Leader-Gag-specific CD8^+^ T-cell responses after preceding immunization with different Env SU-encoding plasmids. CB6F1 mice were immunized once with 25 µg of a plasmid encoding SU Env of F-MuLV, Mo-MuLV, CasBr-MuLV, Hor-MuLV, 4070A-MuLV, FeLV, ALSV, FIV, SIV, or HIV or a control plasmid, followed 3 weeks later by vaccination with 25 µg of pCG.Leader-Gag. Two weeks after the second immunization, the frequency of GagL_85-93_-specific CD8^+^ T cells was analyzed in peripheral blood **(A)**. Three weeks after the second vaccination, the mice were infected with 5,000 SFFU FV by intravenous injection. Three weeks after the FV challenge, the mice were sacrificed and the viral loads in the spleens were determined **(B)**. Data from 8 to 12 (A: 8 to 24) mice per group were acquired in two to three independent experiments. Each dot indicates an individual mouse, and bars indicate mean **(A)** or median values **(B)**. Statistically significant differences compared with the unvaccinated group are indicated by * (*p* < 0.05, Kruskal–Wallis one-way ANOVA on ranks, Dunn’s post-test).

There is no known CD8^+^ T-cell epitope in the F-MuLV Env protein; therefore, we are confident that the observed suppression for this Env SU is not simply due to epitope competition. Since some of the other Env proteins on the other hand do contain CD8^+^ T-cell epitopes that may be recognized in CB6F1 mice, we performed a sequential immunization experiment where mice were first immunized with the Env SU plasmids and subsequently received the Leader-Gag plasmid 3 weeks later. The GagL_85-93_-specific CD8^+^ T-cell responses were comparable to the response observed after two immunizations in the mice that received the Leader-Gag plasmid alone or in combination with a control vector, and still tended to be reduced after the preceding immunization with most Env SU-encoding plasmids. Notably, in this experimental setting, a preceding immunization with FIV, SIV, or HIV Env SU did not impair the GagL_85-93_-specific CD8^+^ T-cell response (**Figure 8A**), suggesting that the strong suppression of CD8^+^ T-cell induction observed before was indeed due to epitope competition. When mice were challenged with FV, we observed less protection from FV infection in most mice compared with the prime-boost immunization performed before (**Figure 8B**). However, some of the mice that received immunization with Env SU of F-MuLV, Moloney-MuLV, 4070A-MuLV, FIV, SIV, and HIV were able to strongly control the FV challenge and had undetectable viral loads in spleens 21 days after FV challenge, which likely reflects a stronger induction of GagL_85-93_-specific CD8^+^ T cells in the mice pre-immunized with the immunodeficiency virus Env SUs or a contribution of (cross-reactive) Env-specific CD4^+^ T cells induced by the murine leukemia virus Env SUs, respectively.

## Discussion

It has long been recognized that retroviral Env proteins have immunosuppressive properties, which result in reduced lymphoproliferative responses and immune activation and altered cytokine profiles (1, 2). While these properties have mostly been described in the context of retrovirus infection, it has also been shown that Env-expressing tumor cells are strongly immunosuppressive (8), and we previously demonstrated that Env can have a severely limiting effect on the induction of CD8^+^ T-cell responses in the context of adenovirus- or DNA-based immunization (15, 16).

Our current findings shed new light on the mechanism underlying the suppression of CD8^+^ T-cell responses by F-MuLV Env. The immunization with an F-MuLV Env plasmid showed that there was no increase in IL-10-producing myeloid cells after immunization, indicating that these cells are not a significant source of IL-10 upon Env stimulation. This adds novel insight to previous findings by Denner et al., who analyzed the cytokine profile of peripheral blood mononuclear cells after incubation with Env TM ISD peptide: the authors reported altered cytokine profiles with increased IL-10 levels but did not analyze the IL-10-producing cell type (1). Interestingly, in humans infected with HIV, increased levels of IL-10 production were also found in T cells, B cells, and NK cells, whereas myeloid cells were found to produce IL-10, but at very similar levels as cells from HIV-uninfected patients (35). In our study, the inactivation of IL-10 in CD4^+^ T cells abrogated the suppression of CD8^+^ T-cell responses, indicating that B and NK cells play only a minor, if any, role in F-MuLV Env-mediated suppression. It is important to note that all DNA preparations used in our experiments were purified by cesium chloride gradient ultracentrifugation, which yielded very pure DNA with very low endotoxin content, making an influence of endotoxin on the observed IL-10 induction highly unlikely.

The co-immunization of CD4-IL-10ko mice, where no Env-mediated suppression was observed, as well as the anti-IL-10 treatment in the context of both the suppression of tumor control by Env SU+TM and the suppression of CD8^+^ T-cell responses by Env SU, clearly shows that mechanistically, an increased IL-10 production by CD4^+^ T cells underlies the Env SU+TM as well as Env SU-mediated suppression. It should be pointed out that the ablation or blocking of IL-10 restored the CD8^+^ T-cell response to the level observed without Env co-administration, rather than increasing it beyond that level. The fact that abrogation of IL-10 production by CD4^+^ T cells alone was sufficient to alleviate suppression of CD8^+^ T cells falls very well in line with previous experiments, where we observed an increased frequency of IL-10-producing CD4^+^ T cells after Env immunization but no increase in regulatory T cells (16), which have been shown to play an important role in controlling CD8^+^ T-cell responses in chronic FV infection (10, 12, 36). The effect of anti-IL-10 treatment on the suppression of CD8^+^ T-cell responses by Env SU was somewhat less robust, and at this stage, we cannot exclude additional factors induced by Env SU influencing CD8^+^ T-cell responses.

As we have observed before, mice that have mounted a low number of GagL_85-93_-specific CD8^+^ T cells are nonetheless able to control FV infection significantly better than unvaccinated mice since the rapid anamnestic expansion of CD8^+^ T cells promotes early control of FV replication and prevents disease development (16, 37). Since most mice that were co-immunized with Env SU plasmids were able to control the FV challenge infection, our results show that the Env-mediated suppression of CD8^+^ T-cell induction reduced the frequency of GagL_85-93_-specific CD8^+^ T cells but left the induced cells functional, responsive, and able to control the FV infection.

The immunization of IL-10-eGFP reporter mice also showed that the frequency of IL-10-producing CD4^+^ T cells increased in both draining and non-draining lymph nodes, showing that the local immunization leads to a systemic effect. This corresponds well with our previous finding that a mere spatial separation of an Env-encoding vector and a vector encoding a CD8^+^ T-cell immunogen would not lead to the rescue of the induction of a CD8^+^ T-cell response (16). Similarly, we showed in our new experiments that the control of a subcutaneous tumor was impaired when mice had received an Env immunization 3 days before. This extends the findings by others that otherwise rejected tumor cells could proliferate in mice when they were stably transduced to express Moloney-MuLV Env (8). Interestingly, we observed the suppressive effect of Env immunization on the control of FBL-3 lymphoma cells, which themselves express F-MuLV antigens including Env (18). The fact that FBL-3 tumor cells are controlled in spite of their Env expression and that Env DNA immunization leads to impaired control suggests that the expression levels determine the degree of IL-10 induction and suppression of CD8^+^ T-cell induction.

The suppressive effect on CD8^+^ T-cell responses was not observed for all tested immunogens, which may be due to the strength of the CD8^+^ T-cell epitopes and the avidity of the CD8^+^ T cells. It is likely that CD8^+^ T cells with lower avidity are more susceptible to IL-10 in the cytokine milieu, and their induction is more easily suppressed. While the Leader-Gag epitope GagL_85-93_ is the immunodominant epitope in FV infection (33), it can easily be dominated by AdV epitopes (37, 38), demonstrating that it is a relatively weak epitope. However, we previously observed that CD8^+^ T cells against the strong ovalbumin epitope Ova_257-264_ were suppressed when an Env-encoding vaccine vector was co-administered with the ovalbumin-encoding vector (16), showing that CD8^+^ T-cell responses against strong immunogens can also be suppressed by Env co-immunization. It is interesting to note that the CD8^+^ T-cell response against F-MuLV Leader-Gag and influenza NP in C57BL/6 mice and against HBsAg in CB6F1 mice tended to be higher after co-immunization with Env than without co-immunization, which may be attributed to the presence of CD4^+^ T helper cell epitopes in Env (39) and to thereby improved CD4^+^ T cell help for the developing CD8^+^ T-cell response. Furthermore, suppression was not observed in all mouse strains, indicating that differentially expressed host factors are likely involved in the suppressive mechanism, which will be addressed in future studies.

Interestingly, early experiments did not indicate a major role of Env SU in immunosuppression, and the focus has since been on the immunosuppressive properties of Env TM. An exception is the HIV SU protein, which has been ascribed immunosuppressive properties (40, 41), but this was explained mechanistically by the occupation of CD4 through Env binding (42). On the other hand, our results clearly show that Env SU contributes to Env-mediated suppression. Compared with earlier co-immunization experiments, it has to be noted that the suppressive effect of F-MuLV Env SU alone delivered by DNA vaccination was somewhat less pronounced than it was observed before for delivery by adenovirus (AdV)-based vectors, where we also observed the suppressive effect more clearly in the AdV-based sequential immunization with F-MuLV Env SU followed by Leader-Gag (16). It is likely that the stronger lasting effect of Env SU in an AdV-based immunization is due to the induction of IL-10-producing AdV-specific CD4^+^ T cells, which hinder the subsequent induction of CD8^+^ T-cell responses against a second immunogen delivered by an AdV-based vector.

We also observed a stronger suppressive effect when we immunized mice with the whole F-MuLV Env delivered by DNA immunization (15). The whole F-MuLV Env includes the TM protein, which contains the immunosuppressive domain described before that has been linked to the induction of altered cytokine secretion and impaired immune cell activation (1, 4, 5), which explains the stronger effects compared with Env SU alone.

Of note, the immunosuppressive effect of Env does not lead to an absence of CD8^+^ T-cell responses in the actual FV infection. On the contrary, GagL_85-93_-specific CD8^+^ T cells are strongly induced and are crucial for the control of an acute FV infection (43). Similarly, the FBL-3 tumor cell line that we used in our tumor experiments expresses Env and Gag, yet mice control the tumor and mount a GagL_85-93_-specific CD8^+^ T-cell response. It may be taken into consideration that the cell types involved in the induction of CD8^+^ T-cell responses may be different in immunization and FV infection and that expression levels affect the quality of the immune response and, therefore, contribute to the different outcomes.

Overall, our experiments indicate that in addition to the Env TM immunosuppressive domain, all gamma retrovirus Env SUs exert a certain degree of suppression on CD8^+^ T cells, which is not observed for the lentivirus Env SUs when used in a sequential immunization. Sequence comparisons show that several regions of the gamma retrovirus Env SUs show a degree of sequence conservation that is similar to that observed for the Env TM immunosuppressive domain (data not shown). It will be important to identify the sequence that is responsible for the suppression by Env SU and identify host factors other than IL-10 that may possibly be involved in mediating the suppression. This may lead to insights into retrovirus pathogenesis and possibly into options for immunotherapeutic approaches. The identification and characterization of the responsible domain in Env SU shall be the aim of further investigation.

## Data availability statement

The raw data supporting the conclusions of this article will be made available by the authors, without undue reservation.

## Ethics statement

The animal study was reviewed and approved by Landesamt für Natur, Umwelt und Verbraucherschutz Nordrhein-Westfalen.

## Author contributions

PP, AM, SW, AP, LK, and DL performed the experiments. MT, ML, MS, KL, WH, and WB provided the resources and methodology and contributed to the conceptualization. PP and WB curated, analyzed, and visualized the data and wrote the manuscript. WB was responsible for the concept of the study and the acquisition of funding. All authors contributed to the manuscript revision and read and approved the submitted version of the manuscript.
