# Supplementary material for: Immune suppression of vaccine-induced CD8+ T-cell responses by gamma retrovirus envelope is mediated by interleukin-10-producing CD4+ T cells
Source: Front Immunol. 2022 Dec 20;13:934399. doi: 10.3389/fimmu.2022.934399 (PMC9807908; doi:10.3389/fimmu.2022.934399)
Supplement: Supplementary file 1 [file DataSheet_1.pdf]

Supplementary Figure 1

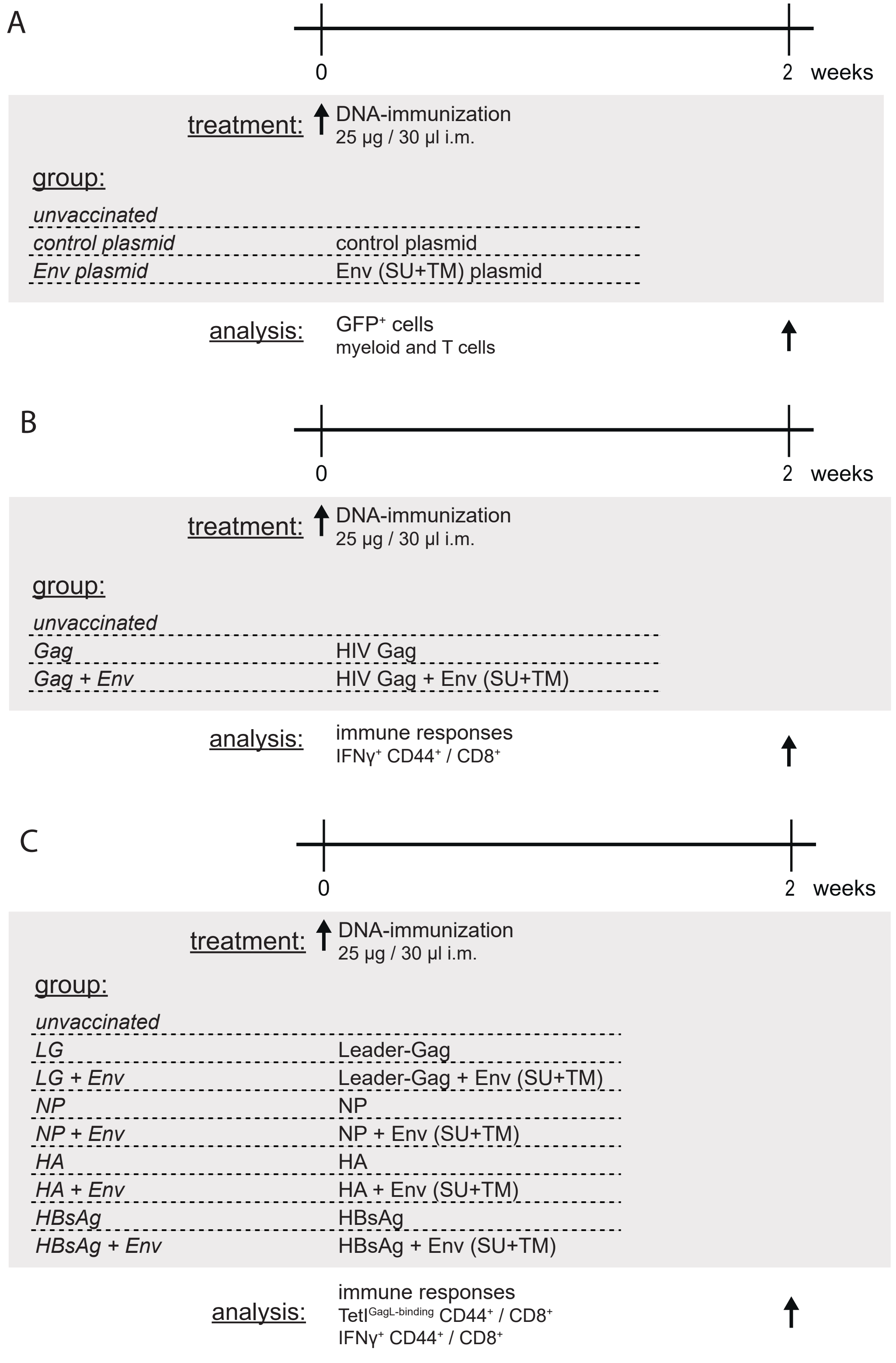

Supplementary Figure 1 (continued)

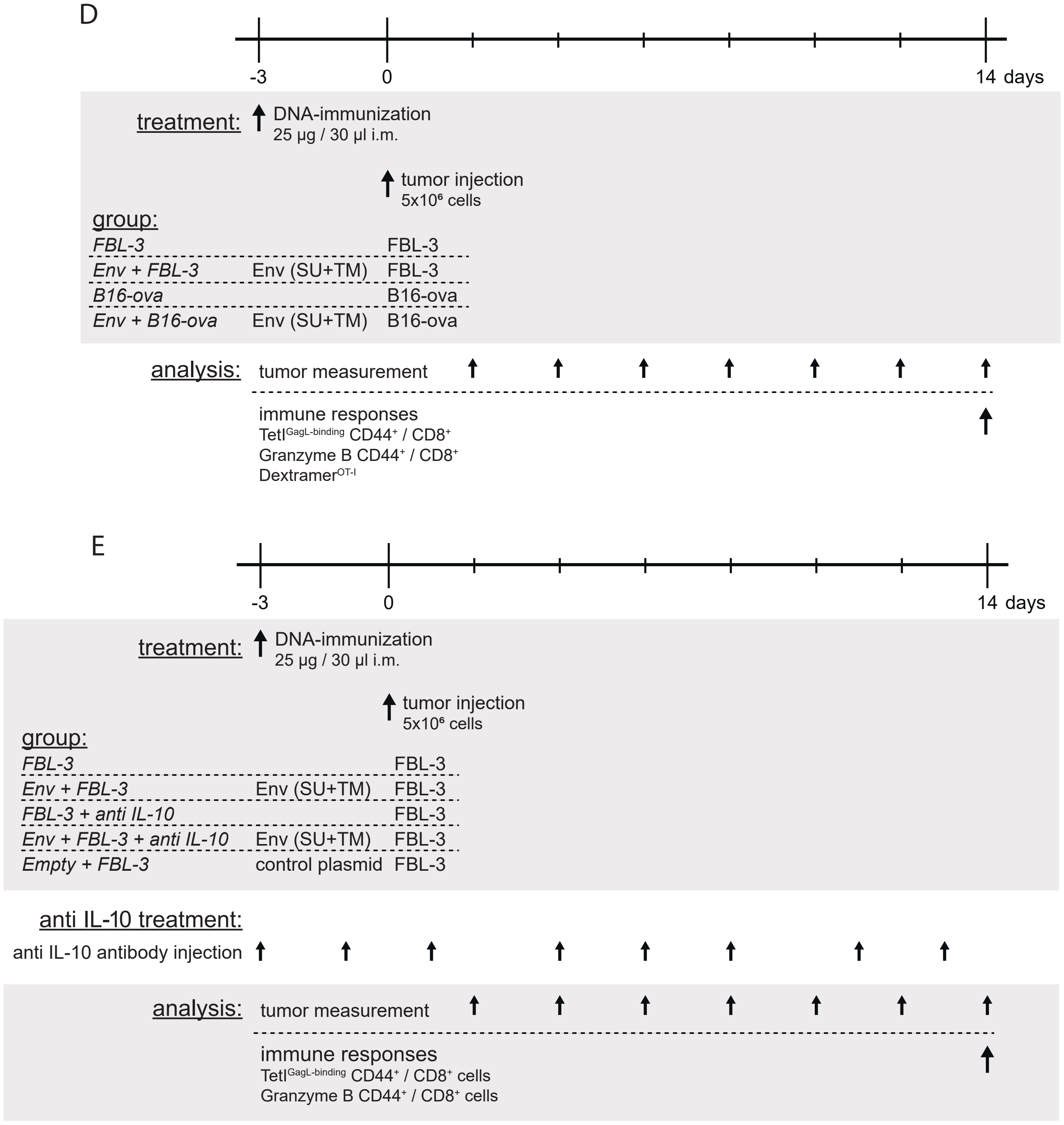

Supplementary Figure 1 (continued)

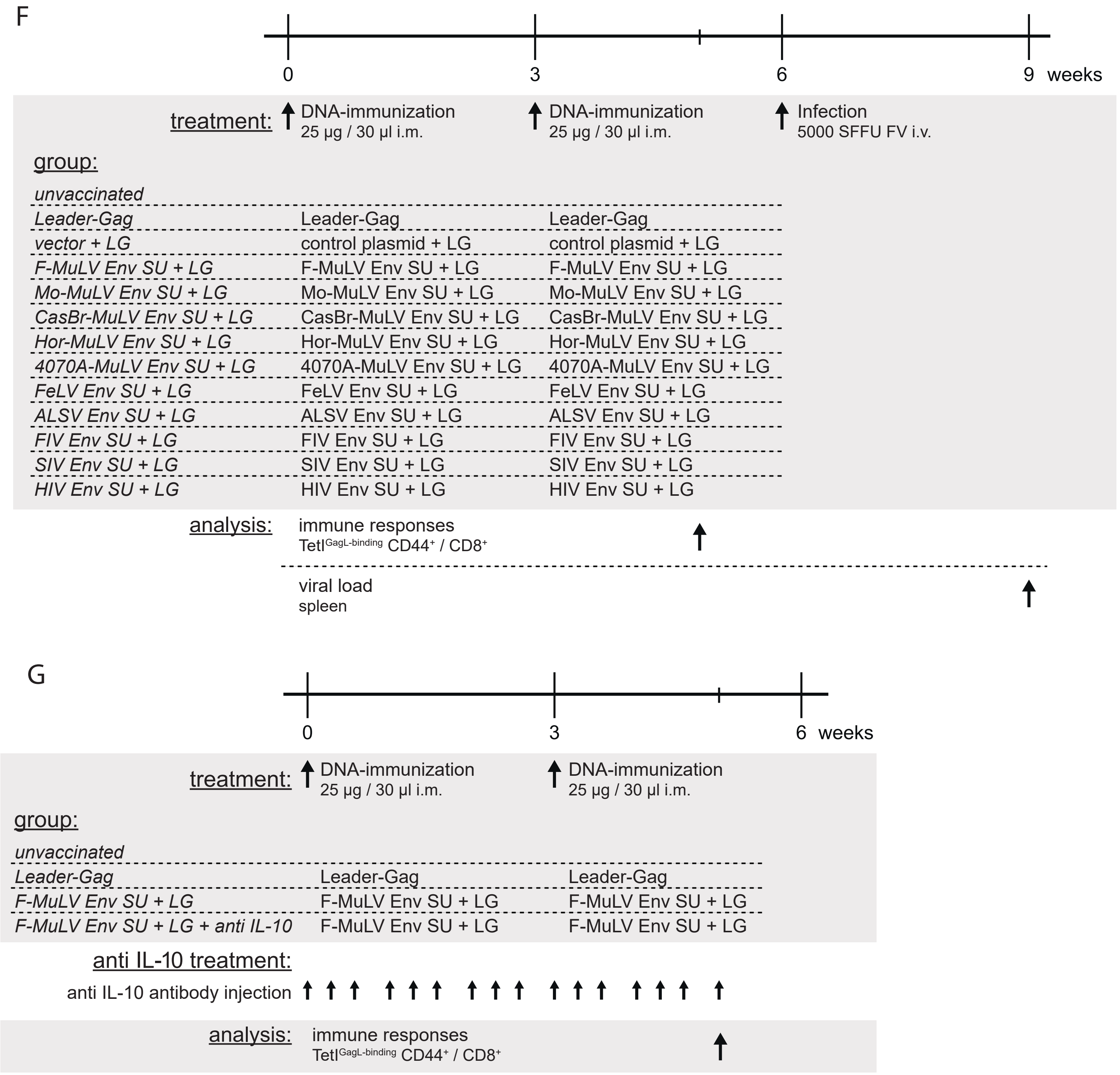

Supplementary Figure 1 (continued)

H

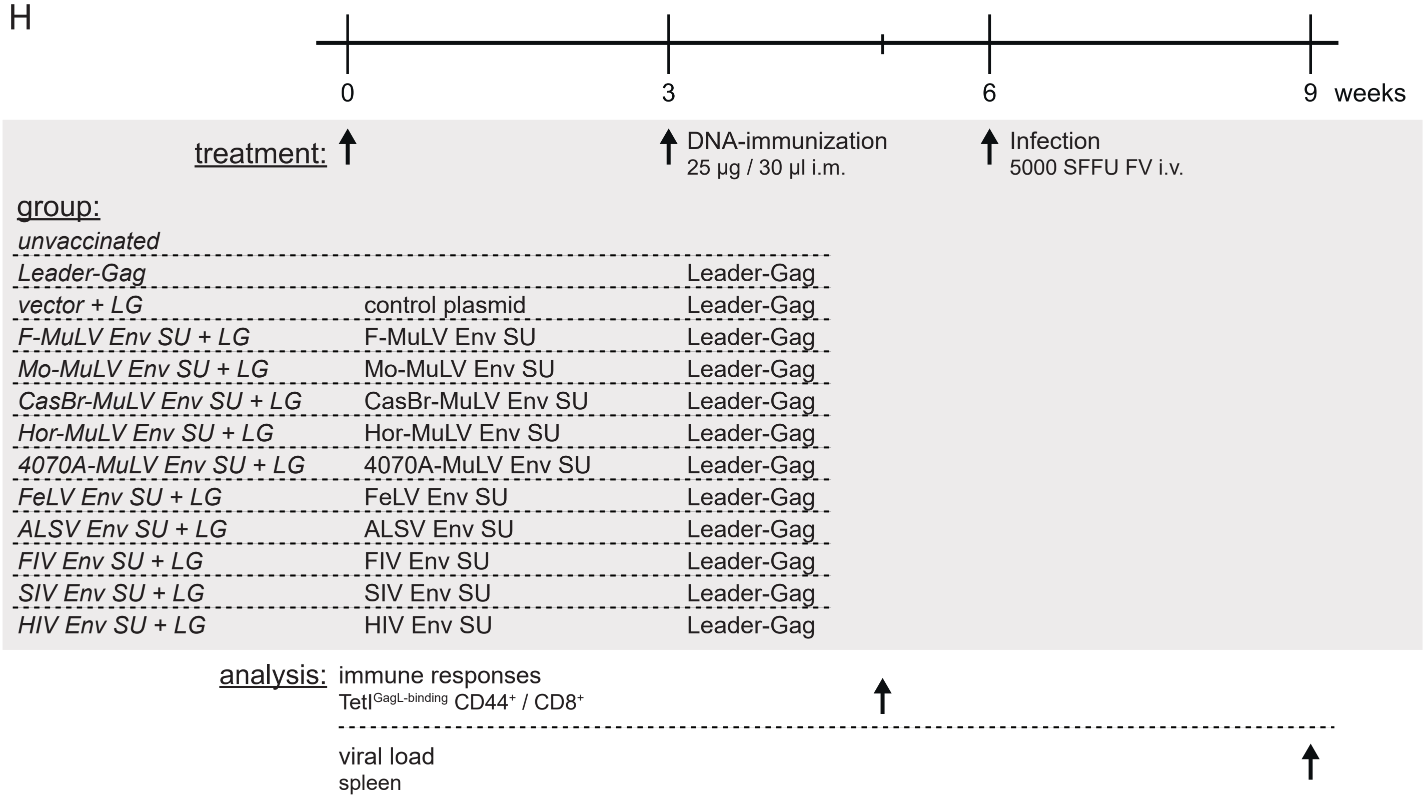

Immunization schemes. (A) IL10-eGFP reproter mice were immunized once with an empty control plasmid or a plasmid encoding F-MuLV Env (SU+TM), eGFP expression of myeloid and T cells was analyzed two weeks later. (B) BALB/c or conditional IL-10ko mice were immunized once with an HIV Gag expression plasmid alone or in combination with an F-MuLV Env (SU+TM) expression plasmid, the frequency of HIV Gag-specific $CD8^{+}$ T cells was analyzed two weeks after immunization. (C) Mice were immunized once with the indicated immunogen encoding plasmids alone or in combination with F-MuLV Env (SU+TM) encoding plasmid, the frequency of HIV Gag-specific $CD8^{+}$ T cells was analyzed two weeks after immunization. (D) Mice were immunized once with an F-MuLV Env (SU+TM) encoding plasmid and inoculated three days later with FBL-3 or B16-ova tumor cells. Tumor size was measured every other day and the frequency of $GagL_{85\text{-}93}$- or $Ova_{257\text{-}264}$-specific $CD8^{+}$ T cells was analyzed two weeks after tumor cell injection. (E) Mice were immunized once with an F-MuLV Env (SU+TM) encoding plasmid or an empty control plasmid and inoculated three days later with FBL-3 or B16-ova tumor cells. Treatment with an anti-IL-10 antibody was performed three times per week starting on the day of immunization; tumor size was measured every other day and the frequency of $GagL_{85\text{-}93}$- or $Ova_{257\text{-}264}$-specific $CD8^{+}$ T cells was analyzed two weeks after immunization. (F) Mice were immunized twice with a Leader-Gag (LG) encoding plasmid alone or in combination with an empty control plasmid or different retrovirus Env SU encoding plasmids. The frequency of $GagL_{85\text{-}93}$-specific $CD8^{+}$ T cells was analyzed two weeks after the second immunization. Mice were challenged with FV three weeks after the second immunization, and the viral load in spleens was analyzed three weeks later. (G) Mice were immunized twice with a Leader-Gag (LG) encoding plasmid alone or in combination with an F-MuLV Env SU encoding plasmid. Treatment with an anti-IL-10 antibody was performed three times per week starting on the day of immunization. The frequency of $GagL_{85\text{-}93}$-specific $CD8^{+}$ T cells was analyzed two weeks after the second immunization. (H) Mice were immunized once with an empty control plasmid or with plasmids encoding different retrovirus Env SU proteins, and three weeks later with an F-MuLV Leader-Gag encoding plasmid. The frequency of $GagL_{85\text{-}93}$-specific $CD8^{+}$ T cells was analyzed two weeks after the second immunization. Mice were challenged with FV three weeks after the second immunization, and the viral load in spleens was analyzed three weeks later.

Supplementary Figure 2

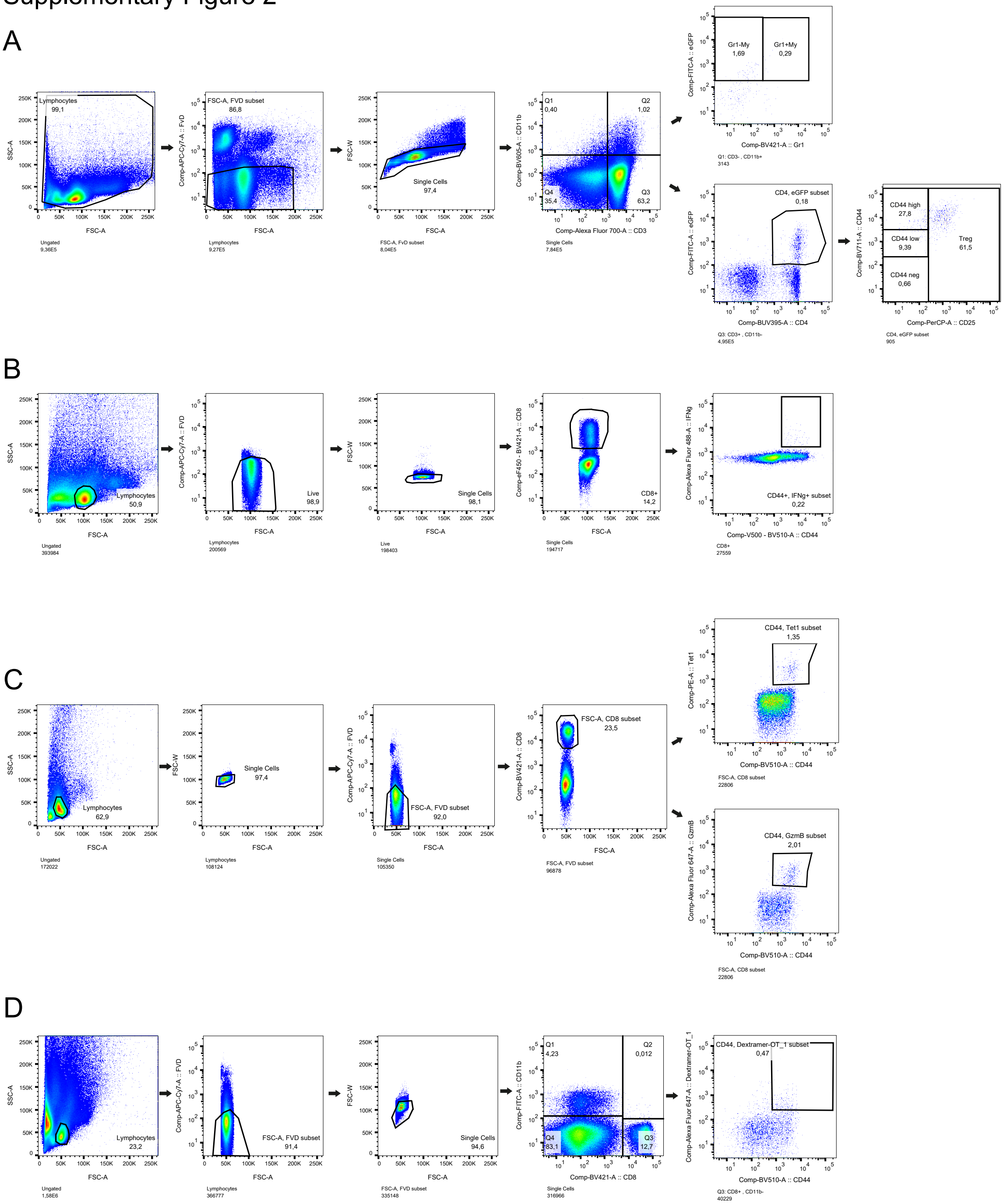

Representative gating schemes. Gating of flow cytometry data was performed by gating on lymphocytes, live cells and single cells. (A) For analysis of eGFP-expressing cell subsets, CD3$^-$ CD11b$^+$ and CD3$^+$ CD11b$^-$ cells were gated and the eGFP expression of Gr1$^-$ and Gr1$^+$ cells (CD3$^-$ CD11b$^+$) or of CD4$^+$ cells (CD3$^+$ CD11b$^-$) was analyzed; CD4$^+$ eGFP$^+$ cells were further gated for CD25 and CD44 expression levels. (B) For the analysis of IFNγ$^+$ cells, single cells were gated for CD8 expression, followed by gating for CD44 IFNγ. (C) For the analysis of GagL$_{85\text{-}93}$-specific CD8$^+$ T cells, single cells were gated for CD8 expression, followed by gating for expression of CD44 and binding of tetramer, or for expression of CD44 and granzmye B. (D) For the analyis of Ova$_{257\text{-}264}$ specific CD8$^+$ T cells, single cells were gated for CD8 expression, followed by gating for expression of CD44 and binding of dextramer.

Supplementary Figure 3

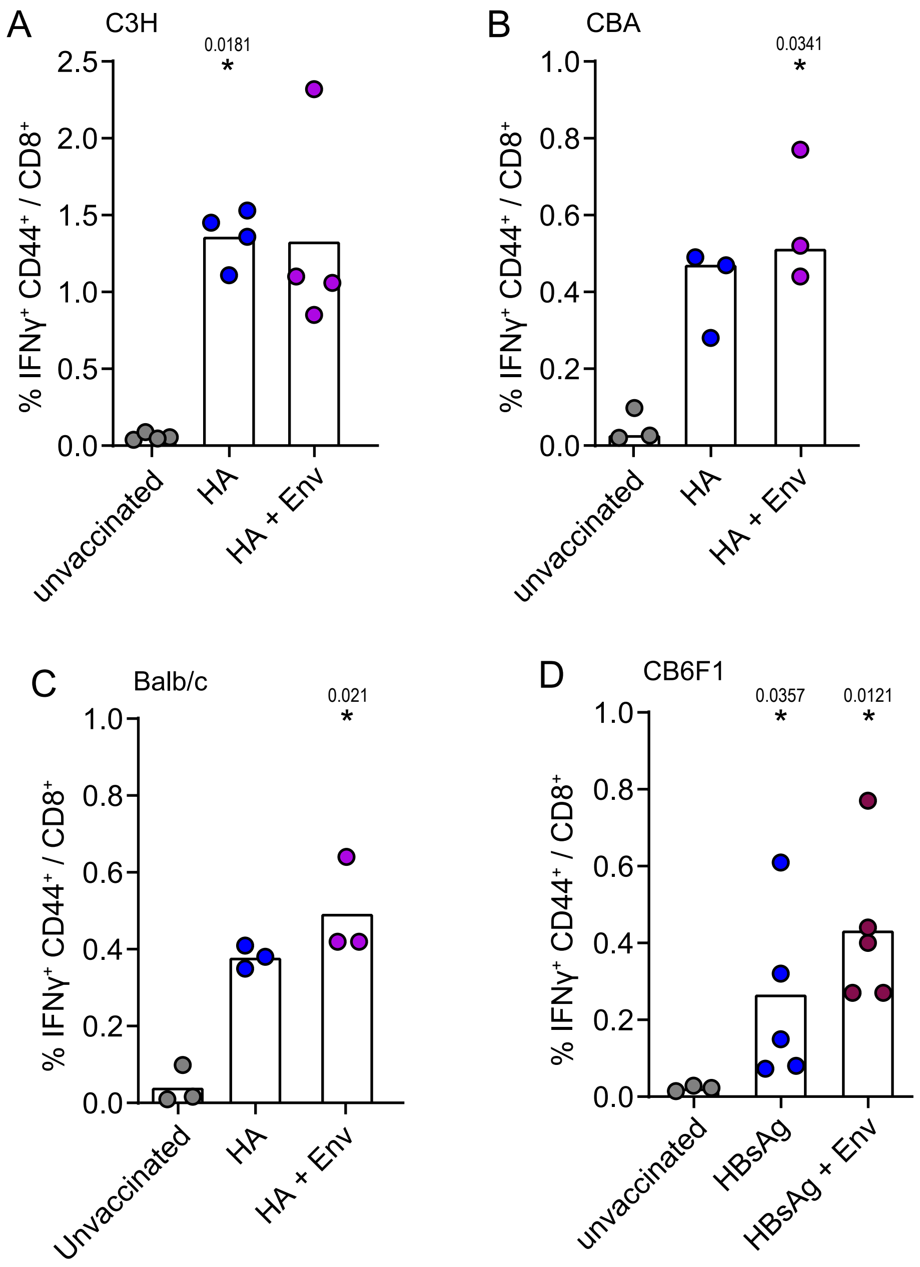

No suppression of influenza hemagglutinin and HBV antigen specific CD8⁺ T cell responses.
C3H(A), CBA (B) and BALB/c mice (C) were immunised once with 25 µg of the influenza virus hemagglutinin encoding plasmid pV.HA alone (HA) or in combination with the F-MuLV Env SU+TM encoding plasmid pCG.env (HA + Env). (D) CB6F1mice (D) were immunised once with 25 µg of a hepatitis B virus surface antigen encoding plasmid alone (HBsAg) or in combination with pCG.env (HBsAg + Env).
Vaccinations were performed by intramuscular injection followed by in vivo electroporation. Two weeks after immunisation, mice were sacrificed and spleens were isolated, and the frequency of immunogen-specific CD8⁺ T cells was determined by intracellular cytokine staining after *in vitro* restimulation with the appropriate peptides.
Data of 3 (B, C), 4 (A) and 5 (D) mice per group were acquired in one experiment, each dot indicates an individual mouse, bars indicate mean values. Statistically significant differences compared to the unvaccinated group are indicated by * ($p < 0.05$, Kruskall Wallis One Way ANOVA on Ranks, Dunn's post test).

Supplementary Table 1 – Statistical analysis of data in Figure 1

			vs. unvaccinated	vs. control
(A) $Gr1^+$ My	draining	control plasmid	0.0323	/
		Env plasmid	0.0241	>0.9999
	non-draining	control plasmid	0.2549	/
		Env plasmid	0.1518	>0.9999
(A) $Gr1^-$ My	draining	control plasmid	>0.9999	/
		Env plasmid	>0.9999	0.6406
	non-draining	control plasmid	>0.9999	/
		Env plasmid	0.4174	>0.9999
(A) $CD4^+$ T	draining	control plasmid	0.2459	/
		Env plasmid	<0.0001	0.0125
	non-draining	control plasmid	>0.9999	/
		Env plasmid	0.0127	0.2891
(B) $CD25^+$ $CD4^+$	draining	control plasmid	0.3176	/
		Env plasmid	0.0049	0.3875
	non-draining	control plasmid	>0.9999	/
		Env plasmid	0.0035	0.1933
(B) $CD44^{high}$ $CD4^+$	draining	control plasmid	0.147	/
		Env plasmid	0.0008	0.2143
	non-draining	control plasmid	0.0633	/
		Env plasmid	0.0218	>0.9999
(B) $CD44^{low}$ $CD4^+$	draining	control plasmid	>0.9999	/
		Env plasmid	<0.0001	<0.0001
	non-draining	control plasmid	0.7066	/
		Env plasmid	0.0036	0.4041
(B) $CD44^-$ $CD4^+$	draining	control plasmid	0.8903	/
		Env plasmid	0.0028	0.0497
	non-draining	control plasmid	0.7081	/
		Env plasmid	>0.9999	>0.9999

Supplementary Table 2 – Statistical analysis of data in Figure 2

		vs. unvaccinated	vs. Gag
(A) Gag pool	Gag	<0.0001	/
	Gag + Env	0.0143	0.0351
(A) $Gag_{192-200}$	Gag	<0.0001	/
	Gag + Env	0.1851	0.0164
(B) BALB/c	Gag	<0.0001	/
	Gag + Env	0.0028	0.0496
(B) CD4-IL10ko	Gag	<0.0001	/
	Gag + Env	<0.0001	0.6312
(B) CD11c-IL10ko	Gag	<0.0001	/
	Gag + Env	0.0093	0.0084

Supplementary Table 3 – Statistical analysis of data in Figure 3

		vs. unvaccinated	vs. w/o Env
(A) CB6F1	LG	0.0006	/
	LG + Env	0.1049	0.3143
(B) C57BL/6	LG	0.3137	/
	LG + Env	0.0035	0.3137
(C) C57BL/6	NP	0.0042	/
	NP + Env	0.0018	>0.9999
(D) C3H	NP	0.075	/
	NP + Env	0.7873	0.7873

Supplementary Table 4 – Statistical analysis of data in Figure 4

day	(A) FBL-3 vs. Env + FBL-3			(B) B16-ova vs. Env + B16-ova		
	tumor size	$TetI^{GagL}$	GzmB	tumor size	Dex^{OT-I}	GzmB
2	0.0001	/	/	0.0933	/	/
4	0.0003	/	/	0.0223	/	/
6	0.0035	/	/	<0.0001	/	/
8	<0.0001	/	/	0.0001	/	/
10	<0.0001	/	/	<0.0001	/	/
12	<0.0001	/	/	<0.0001	/	/
14	<0.0001	0.8996	0.4095	/	0.078	0.01

Supplementary Table 5 – Statistical analysis of data in Figure 5

day	(A) FBL-3 vs. Env + FBL-3	(B) FBL-3 vs. Env + FBL-3 + anti-IL-10	(C) Env + FBL-3 vs. Env + FBL-3 + anti-IL-10	(D) FBL-3 vs. empty + FBL-3	(E) FBL-3 vs. FBL-3 + anti-IL-10
4	<0.0001	<0.0001	0.3571	>0.9999	<0.0001
6	0.0035	0.0502	0.2784	0.0194	0.1319
8	0.0061	0.2913	0.0296	0.6325	0.034
10	<0.0001	0.0082	0.0008	0.611	0.0818
12	<0.0001	0.0045	0.0017	0.3494	<0.0001
14	<0.0001	0.0208	0.0009	0.483	0.0001

		vs. FBL-3	vs. FBL-3 + Env
(F) $TetI^{GagL}$	Env + FBL-3	>0.9999	/
	Env + FBL-3 + anti-IL-10	0.7244	0.6590
	empty + FBL-3	>0.9999	0.8712
	FBL-3 + anti-IL-10	>0.9999	>0.9999
(G) GzmB	Env + FBL-3	>0.9999	/
	Env + FBL-3 + anti-IL-10	0.6911	0.9531
	empty + FBL-3	>0.9999	>0.9999
	FBL-3 + anti-IL-10	0.4914	0.7244

Supplementary Table 6 – Statistical analysis of data in Figure 6

		vs. unvaccinated	vs. Leader-Gag	vs. vector control
(A) TetI^{GagL}	Leader-Gag	0.0003	/	/
	vector + LG	0.0013	>0.9999	/
	F-MuLV Env SU + LG	0.2068	0.5975	0.8858
	Mo-MuLV Env SU + LG	0.0094	>0.9999	>0.9999
	CasBr-MuLV Env SU + LG	>0.9999	0.0003	0.0012
	Hor-MuLV Env SU + LG	0.1498	>0.9999	>0.9999
	4070A-MuLV Env SU + LG	0.5674	0.6473	0.8821
	FeLV Env SU + LG	0.4378	0.8335	>0.9999
	ALSV Env SU + LG	>0.9999	0.0035	0.0102
	FIV Env SU + LG	>0.9999	0.021	0.0467
	SIV Env SU + LG	>0.9999	0.0196	0.044
	HIV Env SU + LG	>0.9999	0.0446	0.0887
(B) infectious centers	Leader-Gag	<0.0001	/	/
	vector + LG	<0.0001	>0.9999	/
	F-MuLV Env SU + LG	<0.0001	>0.9999	>0.9999
	Mo-MuLV Env SU + LG	<0.0001	>0.9999	>0.9999
	CasBr-MuLV Env SU + LG	0.0015	>0.9999	>0.9999
	Hor-MuLV Env SU + LG	0.0630	>0.9999	0.2499
	4070A-MuLV Env SU + LG	<0.0001	>0.9999	>0.9999
	FeLV Env SU + LG	0.0435	>0.9999	0.3344
	ALSV Env SU + LG	0.0022	>0.9999	>0.9999
	FIV Env SU + LG	0.0823	>0.9999	0.1999
	SIV Env SU + LG	0.7046	0.2111	0.0186
	HIV Env SU + LG	0.1910	0.8012	0.0904

Supplementary Table 7 – Statistical analysis of data in Figure 7

		vs. unvaccinated	vs. Leader-Gag	vs. F-MuLV-Env SU + LG
$TetI^{GagL}$	Leader-Gag	<0.0001	/	/
	F-MuLV-Env SU + LG	0.8803	<0.0001	/
	F-MuLV-Env SU + LG + anti-IL-10	0.0015	0.1979	0.0463

Supplementary Table 8 – Statistical analysis of data in Figure

		vs. unvaccinated	vs. LG	vs. vector control
(A) TetI^{GagL}	LG	0.0011	/	/
	vector → LG	0.0454	>0.9999	/
	F-MuLV Env SU → LG	0.1358	>0.9999	>0.9999
	Mo-MuLV Env SU → LG	0.1963	>0.9999	>0.9999
	CasBr-MuLV Env SU → LG	0.3956	>0.9999	>0.9999
	Hor-MuLV Env SU → LG	0.0963	>0.9999	>0.9999
	4070A-MuLV Env SU → LG	0.0552	>0.9999	>0.9999
	FeLV Env SU → LG	0.0737	>0.9999	>0.9999
	ALSV Env SU → LG	0.0762	>0.9999	>0.9999
	FIV Env SU → LG	<0.0001	0.2283	>0.9999
	SIV Env SU → LG	<0.0001	0.2353	>0.9999
	HIV Env SU → LG	<0.0001	>0.9999	>0.9999
(B) infectious centers	LG	>0.9999	/	/
	vector → LG	0.7289	>0.9999	/
	F-MuLV Env SU → LG	0.0015	0.2694	>0.9999
	Mo-MuLV Env SU → LG	0.0093	0.8147	>0.9999
	CasBr-MuLV Env SU → LG	>0.9999	>0.9999	>0.9999
	Hor-MuLV Env SU → LG	>0.9999	>0.9999	>0.9999
	4070A-MuLV Env SU → LG	0.0014	0.2587	>0.9999
	FeLV Env SU → LG	>0.9999	>0.9999	>0.9999
	ALSV Env SU → LG	0.5362	>0.9999	>0.9999
	FIV Env SU → LG	0.0003	0.1057	0.9219
	SIV Env SU → LG	0.0152	>0.9999	>0.9999
	HIV Env SU → LG	0.0005	0.1398	>0.9999

Supplementary Table 9 – Statistical analysis of data in Supplementary Figure 3

		vs. unvaccinated	vs. w/o Env
(A) C3H	HA	0.0181	/
	HA + Env	0.1496	>0.9999
(B) CBA	HA	0.2021	/
	HA + Env	0.0341	0.9121
(C) BALB/c	HA	0.5337	/
	HA + Env	0.021	0.5337
(D) CB6F1	HBsAg	0.0357	/
	HBsAg + Env	0.0121	0.8715
